# Identifying residual hotspots and mapping lower respiratory infection morbidity and mortality in African children from 2000 to 2017

**DOI:** 10.1038/s41564-019-0562-y

**Published:** 2019-09-30

**Authors:** Robert C. Reiner, Catherine A. Welgan, Daniel C. Casey, Christopher E. Troeger, Mathew M. Baumann, QuynhAnh P. Nguyen, Scott J. Swartz, Brigette F. Blacker, Aniruddha Deshpande, Jonathan F. Mosser, Aaron E. Osgood-Zimmerman, Lucas Earl, Laurie B. Marczak, Sandra B. Munro, Molly K. Miller-Petrie, Grant Rodgers Kemp, Joseph Frostad, Kirsten E. Wiens, Paulina A. Lindstedt, David M. Pigott, Laura Dwyer-Lindgren, Jennifer M. Ross, Roy Burstein, Nicholas Graetz, Puja C. Rao, Ibrahim A. Khalil, Nicole Davis Weaver, Sarah E. Ray, Ian Davis, Tamer Farag, Oliver J. Brady, Moritz U. G. Kraemer, David L. Smith, Samir Bhatt, Daniel J. Weiss, Peter W. Gething, Nicholas J. Kassebaum, Ali H. Mokdad, Christopher J. L. Murray, Simon I. Hay

**Affiliations:** 1 Institute for Health Metrics and Evaluation, University of Washington, Seattle, WA USA; 2 Department of Health Metrics Sciences, School of Medicine, University of Washington, Seattle, WA USA; 3 Michigan State University, East Lansing, MI USA; 4 Department of Global Health, University of Washington, Seattle, WA USA; 5 Department of Infectious Disease Epidemiology, London School of Hygiene & Tropical Medicine, London, UK; 6 Department of Zoology, University of Oxford, Oxford, UK; 7 Harvard Medical School, University of Harvard, Boston, MA USA; 8 Imperial College London, London, UK; 9 Big Data Institute, University of Oxford, Oxford, UK; 10 Department of Anesthesiology & Pain Medicine, University of Washington, Seattle, WA USA

**Keywords:** Respiratory tract diseases, Risk factors, Policy and public health in microbiology, Infectious-disease epidemiology, Epidemiology

## Abstract

Lower respiratory infections (LRIs) are the leading cause of death in children under the age of 5, despite the existence of vaccines against many of their aetiologies. Furthermore, more than half of these deaths occur in Africa. Geospatial models can provide highly detailed estimates of trends subnationally, at the level where implementation of health policies has the greatest impact. We used Bayesian geostatistical modelling to estimate LRI incidence, prevalence and mortality in children under 5 subnationally in Africa for 2000–2017, using surveys covering 1.46 million children and 9,215,000 cases of LRI. Our model reveals large within-country variation in both health burden and its change over time. While reductions in childhood morbidity and mortality due to LRI were estimated for almost every country, we expose a cluster of residual high risk across seven countries, which averages 5.5 LRI deaths per 1,000 children per year. The preventable nature of the vast majority of LRI deaths mandates focused health system efforts in specific locations with the highest burden.

## Abstract (web summary)

Geospatial modelling shows an overall decline in morbidity and mortality due to lower respiratory infections in Africa from 2000 to 2017, but also identifies subnational areas with residual high risk.

## Main

Lower respiratory infections (LRIs) are estimated to account for 38.6% of infectious disease deaths and 14.9% of all deaths in African children^1^. These deaths are largely preventable; in particular, reducing risk factors such as household air pollution, stunting and no or partial breastfeeding have been shown to protect children against infection and death resulting from infection^2^. In addition, there are several direct interventions that can prevent or treat infections^3^, including vaccines to the predominant causes of LRI^4,5^, most notably *Streptococcus pneumoniae*, which is estimated to be responsible for 46.7% of LRIs across Africa^6^. Moreover, proven treatments such as antibiotics and supplemental oxygen can prevent death once an individual is infected^3,7^. Although childhood deaths due to LRIs are estimated to have fallen by more than 28.5% in Africa since 2000, this reduction is not geographically uniform, and LRIs still caused more than 432,000 deaths in children under 5 in 2017 (ref. ^1^). In response, the Global Action Plan for the Prevention and Control of Pneumonia and Diarrhoea (GAPPD) of the World Health Organization has called for the trichotomy of ‘protect, prevent and treat’^8^. The immediate need to assemble and analyse all available subnational data on the causes and drivers of LRIs was identified as key to empowering policy aimed at reducing LRI burden.

In 2013, the GAPPD established the goal of reducing child mortality rates attributable to LRIs to below 3 in 1,000 persons and reducing severe LRI episodes to 75% of the 2010 values by 2025 (ref. ^8^). Identifying areas or geographical clusters with the highest residual LRI burden is key to reducing the number of episodes and deaths due to LRI. Of more practical importance, identifying vulnerable populations that are either more likely to become infected by vaccine-preventable aetiologies or more likely to die once infected due to inadequate treatment can optimize targeted intervention strategies. Given the substantial subnational variation in other risk factors and causes of under-5 mortality^9,10^, as well as under-5 mortality itself^11^, country-level estimates of LRI burden probably mask these vulnerable populations. To fully understand the relative local drivers and causes of under-5 mortality, accurately capturing the covariation in risk factors and associated diseases is of paramount importance.

Precision public health focuses on identifying populations in need of specific interventions and providing those interventions in a timely manner^12,13^; as such, spatially and temporally resolved estimates of disease burden are needed to inform targeted interventions. The health burden of LRIs in children is dominated by mortality and not morbidity; thus, reducing LRI burden must ultimately reduce LRI deaths. This starts with geospatial mapping of incidence and mortality of LRIs in children and the risk factors most associated with these diseases. In addition, the case fatality rate (CFR), defined as the ratio of mortality to incidence, is an indicator of treatment-seeking practices and disease management. While some CFR interventions focus on the ‘protect’ component of the GAPPD guidelines, by improving a child’s overall health from birth, a number of more proximal interventions can greatly improve the prognosis of a child’s LRI disease in a clinic or hospital setting^3^. Although national estimates exist^1,2^, policy decisions and interventions rarely occur uniformly at the country level. Similarly, local variation in LRI incidence or mortality may be obscured by estimates restricted to national levels, hindering the ability to identify clusters or hotspots of LRI burden that cross administrative boundaries. While some risk factors for LRIs such as stunting^14^ and no or partial breastfeeding^15^ have been estimated subnationally, with considerable subnational heterogeneity, there are only a few analyses of spatial and spatiotemporal variation in LRI burden within selected regions in Africa^16–18^, and there has not been a comprehensive, subnational analysis of LRI burden for the continent as a whole.

In this study, we present a systematic, comprehensive analysis of local variation in LRI morbidity and mortality in children under 5 across Africa between 2000 and 2017. We use Bayesian model-based geostatistics and an extensive geolocated dataset—describing 9,215,000 LRIs across 1.46 million children from 45,719 total data points, corresponding to 43,080 geolocated point-level survey clusters and 2,639 subnational, geolocated polygons—in combination with established methods from the Global Burden of Disease (GBD) 2017 study^1,19^ to estimate posterior distributions of continuous continent-wide surfaces of LRI prevalence, incidence and mortality. We then use repeated draws from these posterior distributions and aggregate and summarize these to relevant administrative subdivisions (for example, the first administrative level, such as ‘districts’ in Uganda or ‘divisions’ in Kenya; hereafter referred to as ‘divisions’) to identify those with a higher-than-average burden. We juxtapose subnational results with national-level estimates derived from the GBD 2017 to provide further context. Finally, we overlay these results onto detailed analyses of both the percentage of all LRI deaths attributable to *S. pneumoniae* and to LRI CFRs to identify regions of Africa most in need of improved vaccine coverage or case management, respectively.

We conduct out-of-sample tests of model fit and model specification using fivefold cross-validation strategies. At the first administrative level, our estimates closely match the survey data (Supplementary Figs. 15 and 16). A full description of the data, modelling approaches and validation techniques can be found in the Methods. The full output of the analyses is publicly available in the Global Health Data Exchange (http://ghdx.healthdata.org/record/ihme-data/africa-under-5-lri-incidence-prevalence-mortality-geospatial-estimates-2000-2017) and can be explored further via a custom data visualization tool (https://vizhub.healthdata.org/lbd/lri).

## Results

### Substantial subnational heterogeneity in LRI mortality and morbidity through time

**Fig. 1:**
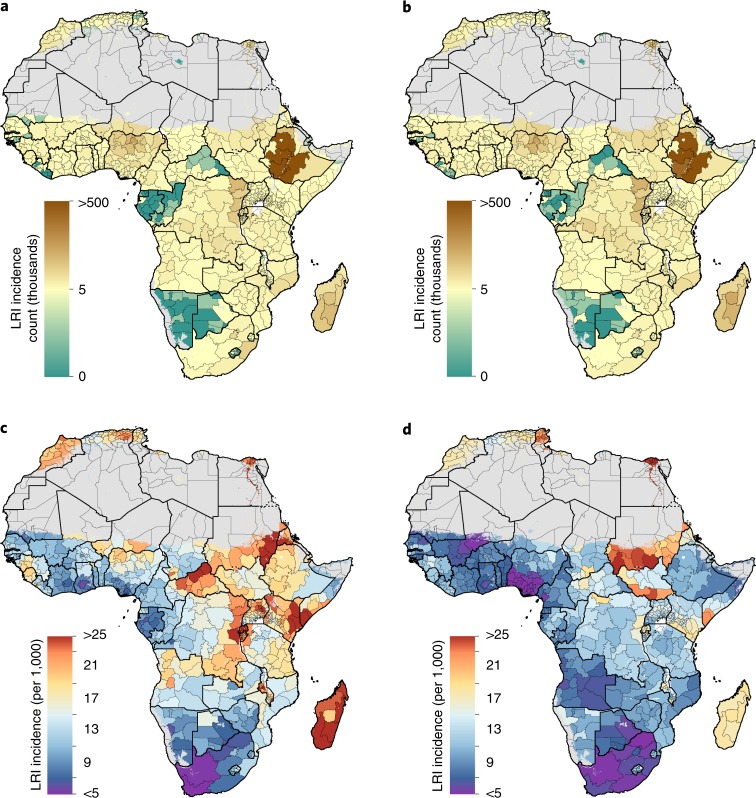
LRI incidence among children under 5 in 2000 and 2017. **a**,**b**, Estimated mean number of LRI episodes aggregated to the first-level administrative subdivision for 2000 (**a**) and 2017 (**b**). **c**,**d**, Estimated LRI episodes rate per 1,000 children aggregated to the first-level administrative subdivision for 2000 (**c**) and 2017 (**d**). Areas with fewer than 10 people per 1 × 1 km^2^ or classified as ‘barren or sparsely vegetated’ are coloured in grey.

In 2017, there were an estimated 23.6 million (95% uncertainty interval (UI) 21.2–27.4) incident LRI episodes in children across all of Africa. Given the 56% increase in the population of children under the age of 5 in Africa, this is a moderate increase in LRI episodes from 20.6 million (UI 19.9–21.3) in 2000 (Fig. 1a,b). The average incidence rate of LRI for African children in 2000 was 165.8 episodes (UI 160.1–171.9) per 1,000, and this rate decreased to 122.2 episodes (UI 109.7–141.6) per 1,000 in 2017 (Fig. 1c,d). According to country estimates derived from the GBD 2017 study, the largest absolute decrease at a national level took place in Angola, where the rate of incident episodes was 165.0 episodes (UI 147.0–183.7) per 1,000 in 2000, decreasing to 83.0 episodes (UI 70.8–96.7) per 1,000 in 2017 (ref. ^6^). The greatest absolute decline in LRI rates among all divisions in Africa occurred in the Blue Nile state, Sudan, from 419.9 episodes (UI 383.0–458.0) per 1,000 in 2000 to 222.1 episodes (UI 62.1–508.5) per 1,000 in 2017 (Fig. 1c,d). In 2017, four countries had greater than threefold variation between divisions: Nigeria; Somalia; Ethiopia; and Senegal. For example, in Nigeria, Yobe had some of the highest rates of LRI episodes across all of Africa in 2017 (148.6 episodes (UI 133.6–164.6) per 1,000), while Anambra had rates less than a quarter of that rate (32.3 episodes (UI 25.2–40.8) per 1,000) (Fig. 1d).

In spite of general declines in incidence across Africa, the GAPPD goal of decreasing severe incidence by 75% of its 2010 values by 2025 is ambitious. It is difficult to evaluate this goal subnationally, since severity data are scarce at the first administrative level. However, under the simplifying assumption that severe incidence is a constant proportion of all incidence across geographies, years, sexes and ages, extremely few divisions are on pace to hit the goal by the 2025 deadline (Supplementary Fig. 2).

**Fig. 2:**
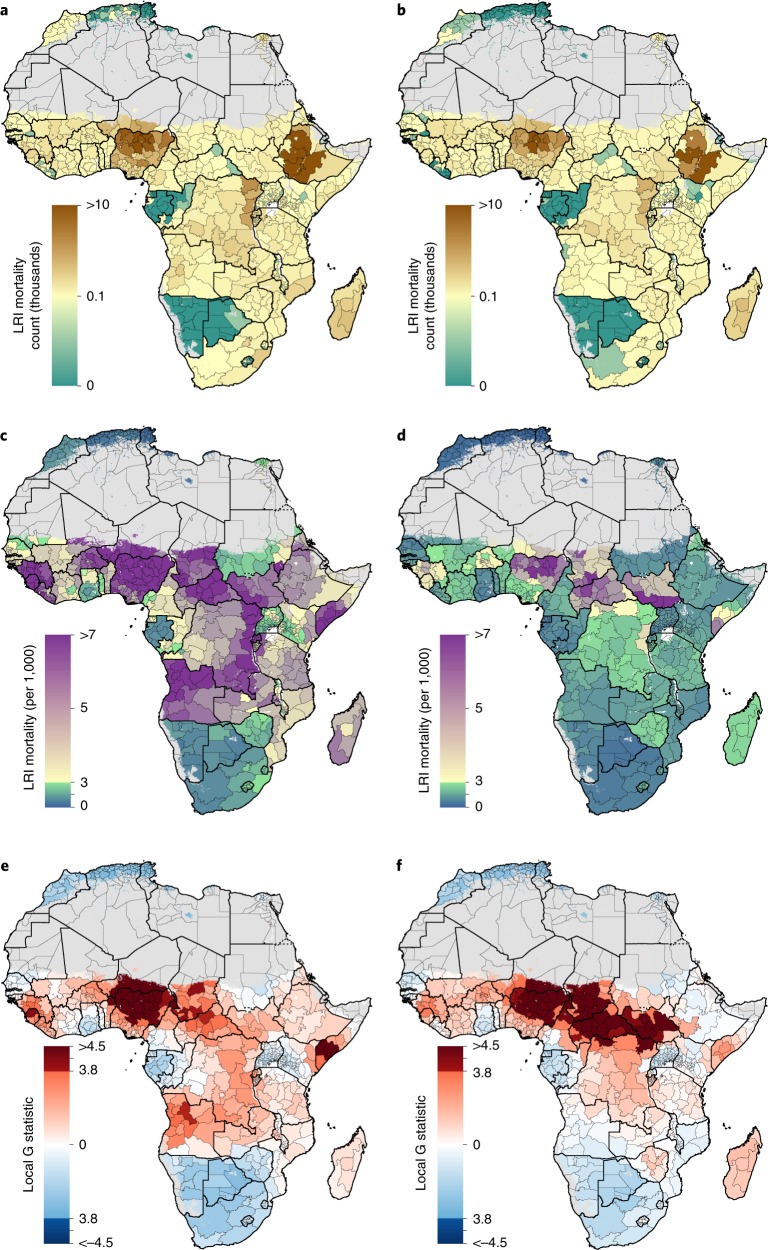
LRI mortality rate, number of deaths and clustering of risk among children under 5 in 2000 and 2017. **a**,**b**, Estimated mean number of LRI deaths aggregated to the first-level administrative subdivision for 2000 (**a**) and 2017 (**b**). **c**,**d**, Estimated mean mortality rate per 1,000 children due to LRI aggregated to the first-level administrative subdivision for 2000 (**c**) and 2017 (**d**). **e**,**f**, *z*-scores as determined by the hotspot analysis for 2000 (**e**) and 2017 (**f**). Discontinuities in the colour scale at −3.8 and 3.8 correspond to locations identified as coldspots or hotspots, respectively (based on adjustments for multiple hypotheses tests). Areas with fewer than 10 people per 1 × 1 km^2^ or classified as ‘barren or sparsely vegetated’ are coloured in grey.

**Fig. 3:**
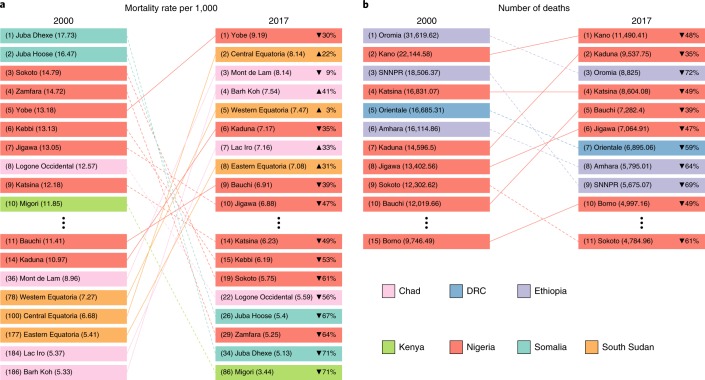
LRI mortality rate and number of deaths among children under 5 by first-level administrative subdivision, 2000 and 2017. **a**, The 10 first-level administrative subdivisions with the highest mortality rates (per 1,000) associated with LRIs in 2000 and 2017. **b**, The 10 first-level administrative subdivisions with the highest number of childhood deaths associated with LRIs in 2000 and 2017. Regions not in the top 10 in both 2000 and 2017 are listed below the vertical ellipses with their associated year-specific rank. The lines connecting regions are solid if the rank increased from 2000 to 2017 and dashed if the rank decreased. Relative change in values is shown for 2017. SNNPR, Southern Nations, Nationalities, and Peoples’ Region.

There were 432,000 (UI 404,000–461,000) LRI deaths estimated among African children in 2017, an average mortality rate of 2.3 (UI 2.1–2.4) deaths per 1,000 (Fig. 2b,d)^1^. This was a substantial decline from numbers in 2000 where the total estimated number of African children who died from LRIs was 604,000 (UI 576,000–636,000) and the average LRI death rate was 4.9 per 1,000 (UI 4.6–5.1) (Fig. 2a,c). Among regions with a mortality rate of at least 2 per 1,000 in 2017, we found increases in 14 divisions from five countries: Burkina Faso (Centre, containing 328,000 children); Chad (Mandoul and Moyen-Chari regions, containing collectively 311,000 children); Central African Republic (Lobaye and Ouham-Pendé prefectures, containing collectively 195,000 children); South Sudan (Central, Eastern and Western Equatoria, containing collectively 660,000 children); and Zimbabwe (Manicaland, Mashonaland West, Masvingo, both North and South Matabeleland and Midlands provinces, containing collectively 1.86 million children) (Fig. 2c,d). Despite a general pattern of decreasing rates, we found that mortality rates were consistently high throughout the study period in the Central African Republic (CAR), Chad, Nigeria and South Sudan (Figs. 2c,d and 3). In 2017, the largest mortality rate difference within a single country’s divisions was observed in Nigeria, with estimates ranging from 1.8 (UI 1.4–2.2) deaths per 1,000 in Anambra to 9.2 (UI 8.1–10.4) deaths per 1,000 in Yobe (Fig. 2d). The largest number of LRI deaths in any divisions in 2017 occurred in Nigeria and Ethiopia; in particular, Kano and Kaduna states in Nigeria (13,800 (UI 12,200–15,500) deaths and 11,500 (UI 9,800–13,300) deaths, respectively) and Oromia state in Ethiopia (12,000 (UI 10,400–13,800) deaths) (Figs. 2b and 3).

In contrast to the severe incidence GAPPD goal, many divisions in Africa appear to have met the GAPPD mortality goal of <3 deaths per 1,000 children (Supplementary Fig. 3). There were 40 countries where every division appeared to have reduced LRI mortality below 3 in 1,000 and 49 countries with at least 1 division at or below the target (Supplementary Table 10). This is in contrast to the situation in 2010, when only 27 countries had completely met the goal and 46 countries had at least 1 division below the threshold (Supplementary Table 10). At the current rate of decline (Supplementary Fig. 3a,b), we expect only 11 countries to have divisions failing to hit the GAPPD mortality goal by 2025 (Supplementary Table 10).

### Concentration of LRI mortality burden

Substantial geographical heterogeneity in LRI mortality risk over time was also apparent from our clustering analyses. Using the Getis–Ord local G statistic, 36 divisions across nine countries (Benin, CAR, Niger, Sierra Leone, Chad, Cameroon, Nigeria, Somalia and Angola) were hotspots of mortality risk in 2000 (Fig. 2e). By 2017, a large, contiguous residual hotspot spanning 54 divisions across seven countries in CAR, South Sudan, Chad, Niger, Cameroon, Democratic Republic of the Congo (DRC) and Nigeria was observed (Fig. 2f). This single, transnational cluster accounted for 30.7% (UI 29.1–32.6) of all child deaths from LRI while including only 13.0% of the population of African children under 5 in 2017. Two sensitivity analyses found that these results were consistent across the posterior distribution of fitted values as well as in settings where all spatial and temporal autocorrelations had been removed from the model fitting process (Supplementary Fig. 6).

Unsurprisingly, countries that were large and consistent contributors to both the 2000 hotspots and residual hotspot in 2017 were those with the most modest relative reductions in mortality rate over the Millennium Development Goal period (1990–2017 reduction: CAR, 17.4% (UI −23.5–46.9); DRC, 52.2% (UI 34.6–64.3); Chad, 42.0%, (UI 20.7–57.8); Nigeria, 59.6%, (UI 46.0–69.7); and South Sudan, 31.9% (UI −1.6–54.3), as derived from the GBD 2017 data). While these reductions are substantial, they are behind the average reduction across Africa over this period (66.0% decrease (UI 51.2–76.7)).

### Potential for targeted interventions to reduce the LRI burden

**Fig. 4:**
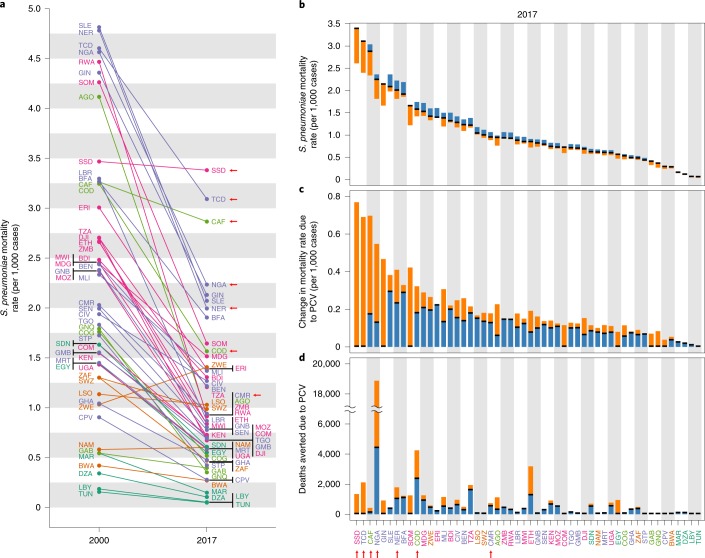
LRI mortality rate attributable to *S. pneumoniae* among children under 5 in 2000 and 2017. **a**, *S. pneumoniae* LRI mortality rate per 1,000 episodes for each country (indicated by the ISO3 abbreviations; www.iso.org/obp/ui) in 2000 and in 2017. **b**, The same mortality rate in 2017 in black, with the blue regions indicating gains made due to current PCV coverage and the orange regions indicating remaining reductions possible with perfect PCV coverage. Countries are plotted in order of the current mortality rate. **c**, The plot from **b** reoriented to illustrate the absolute difference in current mortality rate versus a baseline of no PCV deployment. The height of the orange bar indicates the remaining gain available in mortality rate given 100% PCV coverage. **d**, Same information as **c** but by total number of avertable deaths. Countries in purple are in western sub-Saharan Africa, countries in light green are in central sub-Saharan Africa, countries in orange are in southern sub-Saharan Africa, countries in pink are in eastern sub-Saharan Africa and countries in dark green are in North Africa. The arrows indicate countries located in the 2017 residual hotspot.

Understanding which subnational regions have the highest remaining LRI burden is a first step towards reducing burden. Nearly half of all childhood LRI deaths in Africa are attributable to *S. pneumoniae* (201,000 (UI 99,100–317,000) deaths)^1^, but as with all-cause LRI mortality, the burden of LRIs attributable to *S. pneumoniae* has substantially fallen across almost all of Africa since 2000, with the notable exception of Zimbabwe, where *S. pneumoniae* mortality rates have increased by 40.3% (Fig. 4a). Although the current pneumococcal conjugate vaccines (PCVs) are not perfectly effective and do not cover all *S. pneumoniae* serotypes currently causing mortality and morbidity in Africa^20^, they are still a potent tool to prevent LRIs. While PCV was only introduced in select African countries over the last decade^21^, it is already clear that PCV is contributing to the reduction of burden in the countries where it has been introduced^6^. It is likewise clear that there is still a considerable unmet need for higher PCV coverage. South Sudan has the highest *S. pneumoniae* mortality rate at 3.4 (UI 1.7–5.5) deaths per 1,000 (Fig. 4a) and currently has no PCV programme. Following aetiological fraction calculations from the GBD study^2^ (see Methods for more details), we estimated a 23.5% (UI 14.7–32.4) reduction in this mortality rate is possible with complete childhood PCV coverage, which would avert 0.8 (UI 0.2–1.8) deaths per 1,000 children, or 1,800 (UI 550–4,100) childhood deaths annually—350 (UI 110–790) from the division of Central Equatoria alone (Fig. 4b–d). Conversely, Nigeria has an extremely high LRI burden and a much larger population, but it has a PCV programme, with a country-wide estimated 35.3% (UI 22.1–48.3) coverage of PCV13 (ref. ^22^). This programme is already estimated to be saving 4,400 (UI 2,700–5,900) children per year, but complete coverage (an unlikely scenario) could avert an additional 14,400 (UI 8,600–20,000) deaths per year, reducing the *S. pneumoniae* mortality rate in Nigeria by a further 18.8% (UI 11.4–25.2) (Fig. 4b–d). To date, reliable subnational estimates of PCV coverage are not yet available, but under a conservative assumption that PCV coverage rates in the regions of Nigeria with the highest all-cause LRI burden are at best no higher than the country-wide average, over 3,200 (UI 1,900–4,300) additional childhood deaths per year could be averted in the Yobe and Kaduna states alone with complete childhood PCV coverage (Fig. 4d). Since it is plausible that the regions with the highest LRI burden are also the regions with the lowest PCV coverage, these estimates may drastically underestimate potential vaccine impact.

**Fig. 5:**
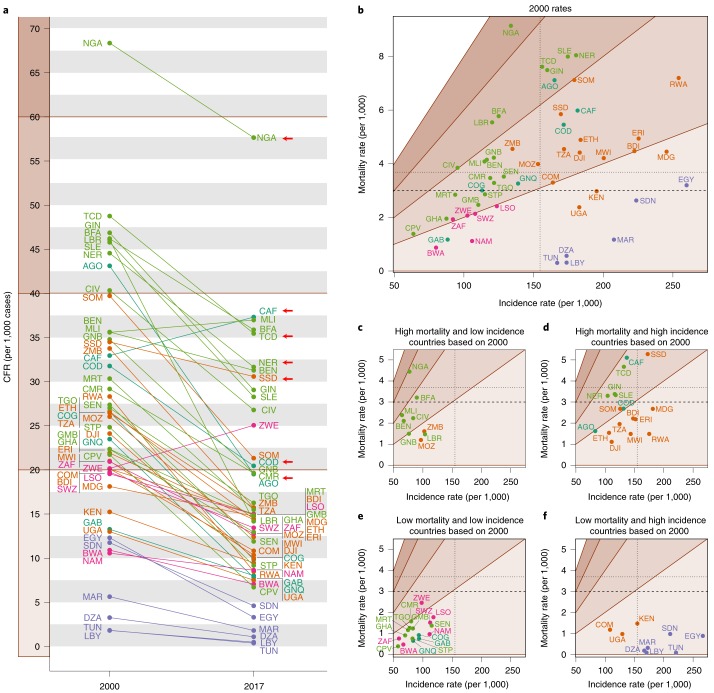
LRI CFR among children under 5 in 2000 and 2017. **a**, LRI CFR per 1,000 cases in 2000 and 2017 for each country (indicated by the ISO3 abbreviations). **b**–**f**, Plots by country and year of incidence rate per 1,000 versus mortality rate per 1,000 for 2000 (**b**) and 2017 (**c**–**f**). The median incidence and mortality rate values are indicated by the dotted lines; the GAPPD goal of 3 deaths per 1,000 children attributable to LRI is indicated by the dashed lines. **c**–**f**, Rates are in the context of how each country performed in 2000 and each panel corresponds to a quadrant of **b**. The ‘high’ and ‘low’ mortality or incidence is defined to be above or below the corresponding median value from 2000. **b**–**f**, The shaded regions from light to dark tan indicate regions of mortality/incidence rate space corresponding to CFRs of 0–20, 20–40, 40–60 and 60–80. The countries in purple are in western sub-Saharan Africa, countries in light green are in central sub-Saharan Africa, countries in orange are in southern sub-Saharan Africa, countries in pink are in eastern sub-Saharan Africa and countries in dark green are in North Africa. The arrows indicate countries located in the 2017 residual hotspot.

As with overall LRI burden, a crucial step in targeting interventions is identifying where LRI CFRs remain highest. Across Africa in 2017, the CFR for childhood LRI was 18.3 (UI 16.3–19.8) per 1,000 and was estimated to be ≥30 per 1,000 in eight countries: Benin; Burkina Faso; CAR; Chad; Mali; Niger; Nigeria and South Sudan, compared to 18 countries in 2000 (Fig. 5a, secondary analysis of the results from the GBD 2017 study). CFRs may obscure the difference between locations where the rates in both the numerator and denominator are high and those where both rates are low; regions of mortality and incidence rate space that result in similar CFRs are indicated by differently shaded regions in Fig. 5b–f. Many countries in the Middle East had low mortality rates in spite of relatively high incidence rates, while in central sub-Saharan Africa many countries had low incidence but relatively high mortality. Both CAR and Mali had a CFR of approximately 38 per 1,000 episodes in 2017 (37.9 (UI 23.8–55.9) per 1,000 for CAR, 37.5 (UI 24.1–53.4) per 1,000 for Mali), but the mortality rate in CAR (5.1 (3.4–7.0) deaths per 1,000) was over twice the mortality rate in Mali (2.4 (1.7–3.2) deaths per 1,000) (Figs. 5a and 2d). Simultaneously, due to substantial difference in the populations of the two countries, the total number of childhood deaths attributable to LRI in Mali (8,890 (7,540–9,300) deaths) was over twice that of CAR (3,090 (2,030–4,270) deaths) (Fig. 2b). In the year 2000, 17 countries were estimated to have incidence and mortality rates that were above the median rates of 154 incident episodes per 1,000 and 3.7 deaths per 1,000 for the continent. By 2017, only South Sudan remained above the 2000 median rates for both mortality and incidence (Fig. 5b–f). In 2017, mortality rates remained above the 2000 median in CAR (5.1 (3.4–7.0) deaths per 1,000), Chad (4.7 (3.6–5.9) deaths per 1,000), Nigeria (4.4 (3.3–5.7) deaths per 1,000) and South Sudan (5.3 (3.9–7.1) deaths per 1,000).

## Discussion

Over the Millennium Development Goal period (1990–2015), dramatic declines at the national scale were documented across Africa, with most countries (42 of 52) reducing their under-5 LRI mortality rate by at least 40%^1^. We found that the rates of decline in incidence and mortality varied both between and within countries across first-level administrative divisions. Some countries had substantial and uniform reductions in LRI burden, while others performed less well in some areas. The divisions that struggled most were clustered into a single region spanning seven countries. Many of these divisions have had consistently high risks over the past 18 years, indicating that they have long been vulnerable. However, identifying the presence (and emergence) of this clustered region represents a unique opportunity for targeting interventions to those in most need. As demonstrated in southern Africa^2,23^, great successes are possible even in high-risk areas.

A recent country-level study found that expanded *Haemophilus influenzae* type b vaccine and PCV coverage and lower exposure to indoor air pollution were the leading independent drivers of declining under-5 LRI mortality in southern sub-Saharan Africa^2^. In Botswana, for example, under-5 LRI deaths declined by an estimated 20.5% due to expanded access to PCV and by 11.0% due to *H. influenzae* type b vaccine coverage^2^. A more in-depth spatiotemporal analysis of the factors that lead to this and other successes could help identify important strategies for reducing both LRI incidence and CFR in the mapped high-risk areas.

The GAPPD framework to ‘protect, prevent and treat’ can guide intervention strategies depending on the local transmission setting. For example, Ethiopia has a below-average CFR, but partially due to its population size, it contains 3 of the top 10 divisions where the most children under 5 die from LRIs (Figs. 5a and 3b). Interventions in Ethiopia should increasingly emphasize prevention, since their treatment rates are already among the best in Africa; however, due to their high population, they still have a considerable LRI burden^22^. By contrast, Nigeria currently has the highest CFR in Africa; thus, interventions that emphasize effective and timely treatment will have a large impact. Nigeria, like a few other countries in central sub-Saharan Africa, has both high CFRs and high incidence rates. As such, interventions focused on preventing infections (such as improved vaccine coverage) would likewise have substantial impact. The local distribution of risk factors—including indoor and outdoor air pollution, child growth failure, poor PCV and *H. influenzae* type b vaccine coverage and inadequate antibiotic use—could be combined with our morbidity and mortality estimates to create locally tailored intervention suites. Critically, in circumstances where increased antibiotic use is recommended, care must be taken to balance optimal intervention for the specific local conditions while simultaneously minimizing risk for an increase in antibiotic resistance^24^.

These results are subject to several limitations. First, to produce continent-wide estimates, we make assumptions about data quality and consistency across a range of sources. For example, LRI prevalence surveys rely on self-reported data on cough and difficulty breathing and are subject to reporting and recall bias; we assume the same level of recall bias across all surveys^25^. Additionally, conversions from prevalence to incidence, estimates of the burden of *S. pneumoniae*, PCV coverage and the CFR analysis all leverage the GBD 2017 study modelled estimates, but GBD assumes that relationships and coverage values are constant within each country. While the conversion from incidence to mortality derived from the GBD study combines various data sources and allows for variation in CFR by country, year, sex and age, it does not currently incorporate the effects of comorbidities. Our geospatial approach borrows strength from neighbouring areas and may smooth over spatially or temporally focal epidemics. Furthermore, while the use of a continuous risk surface is a common approach for determining local risk, accounting for population movement and the clustered nature of populations in rural areas may improve local estimates. Our approach is focused on optimal prediction, not inference, and thus the fitted surfaces are not the optimal tool for assessing the impact of risk factors on risk. Regarding the post-hoc clustering analysis, these approaches, conducted on the modelled surfaces as opposed to the actual data, are used as indices of clustering as opposed to formal statistical tests. In addition, focused clustering analyses could also include more direct clustering approaches^26,27^, but given the coherent nature of the single clustered region identified, it is unlikely that those analyses would yield fundamentally different qualitative conclusions. Finally, there is strong evidence of age-related difference in risk within the 0–5 year age group, especially by aetiology^28,29^. Due to the nature of the data and methods we use, we are currently unable to parse mortality and morbidity estimates into finer age groups or split this burden by aetiology.

The burden of LRI in Africa has fallen dramatically since 2000, but this remarkable progress has not been universal across the continent. The estimates provided in this analysis illustrate the substantial local variability in childhood morbidity and mortality due to LRI, both across Africa and within individual countries. In particular, a single clustered region disproportionately contains much more risk than the rest of Africa. Exemplifying the local variability in risk, no entire country is contained within this region. Rather, subnational portions of countries may be identified as being part of the clustered residual hotspot. As Africa moves towards the 2025 GAPPD goals, those countries that are lagging behind will need specific, detailed focus to make satisfactory progress. The work presented in this study can be combined with targeted interventions to help children in the regions that are most neglected to reduce the mostly avertable burden of LRI.

## Methods

### Overview

For this study, LRIs were defined as diseases of the lower airways including pneumonia and bronchiolitis. Severe LRI episodes, which we present in this study, are those requiring inpatient medical treatment as determined by a physician and based on World Health Organization Integrated Management of Childhood Illness guidelines^30^. Prevalence, incidence and mortality were modelled on continuous continent-wide surfaces and were subsequently aggregated to the first-level administrative subdivisions (referred to as divisions). This study complies with the Guidelines for Accurate and Transparent Health Estimates Reporting recommendations (Supplementary Table 1)^31^. Additional results are provided in the Supplementary Information and online (https://vizhub.healthdata.org/lbd/lri).

### Data sources, standardization and transformations

We compiled 191 household surveys (including the Demographic and Health Surveys, Multiple Indicator Cluster Surveys, World Bank and other country-specific surveys) from 2000 to 2017 with geocoded information from 45,719 total data points, 43,080 corresponding to survey clusters and 2,639 to subnational polygon boundaries. We included surveys that asked about 2- or 4-week prevalence of cough with difficulty breathing among children either under 3 or under 5 years old and allowed for geolocation below the country level. The prevalence of acute respiratory infection symptoms was adjusted to meet a standard case definition for LRIs (clinician-diagnosed pneumonia or bronchiolitis). The transformation of seasonally adjusted prevalence data from period to point estimates is described in detail in the GBD 2016 study of global, regional and national LRI mortality and morbidity^2^.

We differentially scaled our continuous continent-wide surface estimates of the spatial pattern of prevalence to national-level estimates of LRI prevalence and incidence from the GBD 2017 study by calculating a population-weighted mean such that the national estimates presented in this study match those from the GBD 2017 study. To produce the severe incidence estimates, we multiplied our prevalence estimates by the GBD fraction of LRI episodes categorized as severe divided by average duration. CFRs by country and year were obtained from the GBD by dividing the country-specific mortality of LRI in a given year by the country-specific total incidence in that year. Finally, we estimated our continuous continent-wide surface of mortality by multiplying our incidence surface by the appropriate country-year CFRs. Detailed data processing descriptions are provided in Section 2.0 of the Supplementary Information.

### Estimation of spatially explicit LRI prevalence and clustering

Adjusted point prevalence data were used as inputs to a Bayesian model-based geostatistical framework (described in detail in Section 3.3 in the Supplementary Information)^2,19^. Briefly, this framework uses a spatially and temporally explicit hierarchical logistic regression model to predict LRI prevalence in locations with sparse observations where points that are closer together in space and time—and which have similar covariate patterns—are expected to have similar LRI prevalence. Because the prevalence of LRIs has been shown to be influenced by social, structural and environmental factors^2,32^, we selected a set of 12 continuous geographical covariates based both on previous mapping efforts and past GBD analyses of LRI for inclusion in the model (Supplementary Table 3)^33^. Potential non-linear relationships between these covariates and LRI prevalence were incorporated through the use of a stacked generalization technique^33^, which is further detailed in Section 3.2 of the Supplementary Information. Sensitivity analyses were carried out to assess sensitivity to hyper-prior specification and are described in detail in Section 5.4 of the Supplementary Information. For the prevalence estimates in 2017, due to data limitations, we assumed the spatial pattern matched that of 2016. Estimated prevalence rates were converted into incidence rates using an average duration of an episode of LRI of 7.8 d. Using country- and year-specific CFRs from the GBD 2017 study, we converted our incidence rates into mortality rates. Posterior distributions of all model parameters and hyperparameters were estimated using the statistical package R-INLA v.18.07.12 (ref. ^34,35^). Uncertainty was calculated by taking 1,000 draws from the posterior joint distribution of the model; each point value is reported with an uncertainty interval that represents the 2.5th and 97.5th percentiles of those 1,000 draws. Additional methodological details can be found in the Supplementary Information.

We used two post-hoc approaches to identify regions or clusters of higher-than-average burden from our estimates: the Getis–Ord local G statistic^36^ and a simple arithmetic method. The Getis–Ord local G statistic, which in this study relates each first-level administrative subdivision and its neighbours to the total set of first-level administrative subdivisions, identifies whether a division is collectively significantly higher (hotspot) or lower (coldspot) than the expected value as informed by the total set^36^. We then conducted a sensitivity analysis to determine the impact of the spatial and temporal correlations structures inherent to the Bayesian geostatistical approach on the clustering hotspot analysis. Moreover, we conducted the same analysis on each draw from the posterior distribution of mortality risk and summarized the frequency for which each first administrative division was identified as a ‘hotspot’. This sensitivity analysis avoids the use of the standard reference distribution for the local statistic and as such is adjusting for the spatial variation in the background population size. The second method identified clusters of higher-than-average burden arithmetically by identifying all divisions with the highest estimated mortality rate that account for a fixed percentage of all deaths and then counting the number of contiguous divisions identified. It is important to note that for both analyses, ‘hotspot’ is a relative term, comparing the risk in one area to other areas within a single year. Overall burden has decreased from 2000 to 2017, so locations that are identified as hotspots in 2017 would not have qualified in 2000. To emphasize this, when identifying clusters of remaining high risk in 2017, we refer to them as residual hotspots.

### Reporting Summary

Further information on research design is available in the Nature Research Reporting Summary linked to this article.

## Supplementary information

Supplementary Information Supplementary Guidelines, Supplementary Results, Supplementary Figs. 1–20, Supplementary Tables 1–13 and Supplementary References.

Reporting Summary

## Data Availability

The findings of this study are supported by data that are available in public online repositories, data that are publicly available upon request from the data provider and data that are not publicly available due to restrictions by the data provider and were used under licence for the current study. A detailed table of data sources and availability can be found in Supplementary Tables 2 and 3.

## Code availability

All code used for these analyses is publicly available online at http://ghdx.healthdata.org/record/ihme-data/africa-under-5-lri-incidence-prevalence-mortality-geospatial-estimates-2000-2017.
