# Supplementary material for: Identifying residual hotspots and mapping lower respiratory infection morbidity and mortality in African children from 2000 to 2017
Source: Nat Microbiol. 2019 Sep 30;4(12):2310–8. doi: 10.1038/s41564-019-0562-y (PMC6877470; doi:10.1038/s41564-019-0562-y)
Supplement: Supplementary file 1 — Supplementary Guidelines, Supplementary Results, Supplementary Figs. 1–20, Supplementary Tables 1–13 and Supplementary References. [file 41564_2019_562_MOESM1_ESM.pdf]

nature microbiology

SUPPLEMENTARY INFORMATION

https://doi.org/10.1038/s41564-019-0562-y

In the format provided by the authors and unedited.

Identifying residual hotspots and mapping lower respiratory infection morbidity and mortality in African children from 2000 to 2017

Robert C. Reiner[1,2*], Catherine A. Welgan[1], Daniel C. Casey[1], Christopher E. Troeger[1], Mathew M. Baumann[1], QuynhAnh P. Nguyen[1], Scott J. Swartz[1], Brigette F. Blacker[1], Aniruddha Deshpande[1], Jonathan F. Mosser[1], Aaron E. Osgood-Zimmerman[1], Lucas Earl[1], Laurie B. Marczak[1], Sandra B. Munro[1], Molly K. Miller-Petrie[1], Grant Rodgers Kemp[1,3], Joseph Frostad[1], Kirsten E. Wiens[1], Paulina A. Lindstedt[1], David M. Pigott[1,2], Laura Dwyer-Lindgren[1,2], Jennifer M. Ross[4], Roy Burstein[1], Nicholas Graetz[1], Puja C. Rao[1], Ibrahim A. Khalil[1,2], Nicole Davis Weaver[1], Sarah E. Ray[1], Ian Davis[1], Tamer Farag[1], Oliver J. Brady[5], Moritz U. G. Kraemer[6,7], David L. Smith[1,2], Samir Bhatt[8], Daniel J. Weiss[9], Peter W. Gething[9], Nicholas J. Kassebaum[1,10], Ali H. Mokdad[1,2], Christopher J. L. Murray[1,2] and Simon I. Hay[1,2*]

[1]Institute for Health Metrics and Evaluation, University of Washington, Seattle, WA, USA. [2]Department of Health Metrics Sciences, School of Medicine, University of Washington, Seattle, WA, USA. [3]Michigan State University, East Lansing, MI, USA. [4]Department of Global Health, University of Washington, Seattle, WA, USA. [5]Department of Infectious Disease Epidemiology, London School of Hygiene & Tropical Medicine, London, UK. [6]Department of Zoology, University of Oxford, Oxford, UK. [7]Harvard Medical School, University of Harvard, Boston, MA, USA. [8]Imperial College London, London, UK. [9]Big Data Institute, University of Oxford, Oxford, UK. [10]Department of Anesthesiology & Pain Medicine, University of Washington, Seattle, WA, USA. *e-mail: bcreiner@uw.edu; sihay@uw.edu

Supplementary Information: Identifying residual hot-spots and mapping lower respiratory infection morbidity and mortality in African children from 2000 to 2017

Robert C. Reiner†, Catherine A. Welgan, Daniel C. Casey, Christopher E. Troeger, Mathew M. Baumann, QuynhAnh P. Nguyen, Scott J. Swartz, Brigette F. Blacker, Aniruddha Deshpande, Jonathan F. Mosser, Aaron E. Osgood-Zimmerman, Lucas Earl, Laurie B. Marczak, Sandra B. Munro, Molly K. Miller-Petrie, Grant Rodgers Kemp, Joseph Frostad, Kirsten E. Wiens, Paulina A. Lindstedt, David M. Pigott, Laura Dwyer-Lindgren, Jennifer M. Ross, Roy Burstein, Nicholas Graetz, Puja C. Rao, Ibrahim A. Khalil, Nicole Davis Weaver, Sarah E. Ray, Ian Davis, Tamer Farag, Oliver J. Brady, Moritz U. G. Kraemer, David L. Smith, Samir Bhatt, Daniel J. Weiss, Peter W. Gething, Nicholas J. Kassebaum, Ali H. Mokdad, Christopher J. L. Murray & Simon I. Hay†.

†Corresponding authors

Table of Contents

0.0 Guidelines for Accurate and Transparent Health Estimates Reporting (GATHER) Compliance 3

1.0 Case definition of modelled outputs 3

1.1 Prevalence 3

1.2 Incidence 3

1.3 Mortality 3

2.0 Data 3

2.1 Summary of included data sources 3

2.2 Standardising case definitions 3

2.3 Seasonality Adjustment 4

2.4 Period prevalence to point prevalence conversion 4

2.5 Aggregation to finest possible geography 4

2.6 Creation of pseudo-points within areal units 4

2.7 Assigning covariates to points 5

2.8 Administrative Boundaries 5

3.0 Geostatistical Model 5

3.1 Model Geographies 5
3.2 Ensemble covariate modelling via stacked generalisation 5
3.4 Priors 7
3.5 Mesh Creation 7
3.6 Fitted parameters and estimate generation 8
4.0 Post-estimation 8
4.1 Calibration to the Global Burden of Disease (GBD) 2017 8
4.2 Conversion of point prevalence to other measures 8
4.3 Fractional pixel aggregation 9
4.4 Pneumococcal pneumonia attributable fraction 9
5.0 Model Validation 10
5.1 In-sample Validation 10
5.2 Out-of-Sample Validation 10
5.3 Variations of model specification 11
5.4 INLA prior sensitivity analysis 11
6.0 Supplemental Results 12
6.1 Prevalence, incidence, and mortality due to LRI 12
6.2 Global Action Plan for Pneumonia and Diarrhea (GAPPD) projections 12
6.3 Concentration of LRI mortality 12
4.0 Supplementary Figures 14
5.0 Supplementary Tables 42
6.0 Supplementary Information References 65

0.0 Guidelines for Accurate and Transparent Health Estimates Reporting (GATHER) Compliance

Supplementary Table 1 provides the Guidelines for Accurate and Transparent Health Estimates Reporting (GATHER) compliance checklist.

1.0 Case definition of modelled outputs

1.1 Prevalence

We used "clinician confirmed pneumonia or bronchiolitis" as the gold-standard case definition of lower respiratory infection (LRI) with an assumed duration of 7.79 days (95% credible interval (CI) 6.2–9.6)[1].

1.2 Incidence

We reported only incidence of severe LRI. A random effects meta-analysis conducted for the Global Burden of Disease study 2017 (GBD 2017) found that 15.1% (95% CI 13.3–16.9) of all cases of LRI were severe. This ratio is used in the present study[1].

1.3 Mortality

We used the LRI mortality estimates produced by GBD 2017. Specifically, LRI deaths were coded as 073·0-073·6, 079·82, 466-469, 480-489, 513·0, or 770·0 for the ICD9 version and A48·1, J09-J22, J85·1, P23-P23·9, or U04 for ICD10. For additional discussion of how LRI mortality was estimated and how the coding schemes were used refer to GBD 2016[1].

2.0 Data

2.1 Summary of included data sources

The household surveys used to model LRI prevalence are listed in Supplementary Table 2 and visualised in Supplementary Figure 7. For a survey to be considered for this analysis, we required information on whether a child had been coughing recently and had difficulty breathing. Ideal datasets captured additional information about symptoms in the chest and fever status. Select data sources were excluded from the analysis because of: missing survey weights for areal data, incomplete sampling (e.g., only a specific age range), or untrustworthy data (as determined by the survey administrator or by inspection).

2.2 Standardising case definitions

We used joint presence of cough, difficulty breathing, symptoms in the chest, and fever as the preferred definition of LRI from our survey data. For observations for which only partial information was available, we applied a series of adjustments. First, to adjust for imperfect specificity in the survey definitions of LRI, we used a reference survey definition of cough with difficulty breathing with symptoms in the chest and fever. Data from surveys that did not capture information on chest symptoms and/or fever were adjusted using a scalar of the mean

prevalence in the non-reference to the reference survey definition. Subsequently, in order to account for the imperfect specificity of self-reported symptoms, all data were adjusted using a scalar of the ratio of clinician-diagnosed pneumonia or bronchiolitis to the mean prevalence of cough with difficulty breathing and symptoms in the chest with fever. We used a logistic regression to find a conversion factor between LRI prevalence with and without fever symptoms while adjustments accounting for the lack of information on difficulty breathing were calculated using the Bayesian meta-regression tool Dismod 2.1[2]. All survey data were adjusted to the gold-standard case definition (Section 1.1). Supplementary Table 8 lists these adjustments.

2.3 Seasonality Adjustment

Because surveys are rarely conducted over the entire year, estimates of LRI may be biased by seasonal trends. We accounted for intra-annual variation in LRI prevalence by fitting a sine-cosine regression with a period of twelve months by region, weighted by the standard error of the data. We generated and applied a scalar per month and region based on the percent difference between the regression fit and observed LRI prevalence to adjust for seasonal biases (Supplementary Figure 21).

2.4 Period prevalence to point prevalence conversion

Data were converted from period prevalence (e.g., "did child x have y symptoms in the last z days?") to point prevalence using the following formula:

$$Point\ Prevalence = \frac{Period\ Prevalence\ *\ Duration}{(Recall\ Period + Duration - 1)}$$

Where duration is assumed to be 7.79 days and recall period is the number of days the question asks over (e.g., 2 weeks).

2.5 Aggregation to finest possible geography

We aggregated/summarised the individual-level microdata to the finest possible spatial resolution available— preferably, a latitude and longitude pair representing the location of the survey cluster/primary sampling unit. Where point-level referencing was not available, we matched survey microdata to the smallest polygon/areal unit possible. We calculated the effective sample size for each spatial aggregation (point and polygon) via the Kish approximation considering the underlying complex survey design[3]. After aggregation, the adjustments described above (Section 2.2–2.4) were applied.

2.6 Creation of pseudo-points within areal units

We created pseudo-points for areal data via a population-weighted resampling processes as our desired model requires data of a single geometric type (e.g., latitude/longitude point). Specifically, we randomly generated 10,000 candidate points from within each areal unit using the WorldPop total population raster as a spatial distribution weight[4]. K-means clustering was performed to aggregate candidate points into the pseudo-points used for modelling. These pseudo-points were assigned analytical weights proportional to the number of candidate points

that entered into the k-means cluster. Each pseudo-point generated by this process was assigned the LRI prevalence observed from the survey for that polygon.

2.7 Assigning covariates to points

We assembled a number of remotely sensed and modelled products to use as predictors. Where possible we selected covariates that were used to model the burden of LRI for GBD 2017, existed at the temporal (yearly) and spatial resolution of interest (5 × 5 km), and did not exhibit collinearities above .8 with other covariates. Supplementary Figure 8 displays the final selection of covariates while Supplementary Table 3 lists the source information. Once assembled, we conducted a spatial query to match covariate values spatially and temporally to our collection of points and pseudo points. For numerical stability, all covariates were centered and scaled to mean 0, with a standard deviation of 1.

2.8 Administrative Boundaries

All country-level and first-, and second-administrative level boundaries used in this analysis came from the Database of Global Administrative Areas (GADM) version 3.6. GADM shapefiles are available to download from https://gadm.org/. Slight adjustments to ensure proper nesting of administrative units were made, but the boundaries remain unchanged.

3.0 Geostatistical Model

3.1 Model Geographies

We stratified our data and analyses into five contiguous regions selected to align with the GBD study. This was done to improve computational tractability and to take advantage of the a-priori grouping based on country-level epidemiological profiles. Supplementary Figure 9 shows the configuration of the regions.

3.2 Ensemble covariate modelling via stacked generalisation

We used a stacked generalisation ensemble model framework to capture non-linear effects and complex interactions among our covariates[5]. For each region (see section 3.1), we fit four child models to our dataset: a generalised additive model (GAM), a penalised regression with the elastic net penalty, and two boosted regression trees (BRT). As described below in section 3.3, we use a spatio-temporal Gaussian process regression as the parent ensembler.

Parameters for the GAM model (spline type and number of knots) was selected by an expert a priori while the lambda parameter for the elastic net regression was selected by cross-validation. For the BRTs, a coarse grid search was conducted across all the data (same parameters for each region strata) to identify optimal hyperparameters. From the search, two sets of parameters were generated. The first set featured the lowest average root mean squared error (RMSE) while the second set was selected by finding the least correlated alternative with similar out-of-sample validity statistics based on visual inspection. Additional details on the tradeoff between model parsimony and the number of BRT child models can be found in section 5.3.

Each child model was fit using five-fold cross-validation to reduce overfitting and the out-of-sample predictions across the child model hold outs were compiled into a single set of model predictions. Additionally, each child model was fit on 100% of the data and a full set of in-sample predictions were created. The out-of-sample predictions per child model were fed to the parent geostatistical model (see below) as covariates for fitting while the in-sample predictions from the child models are used during the parent model's predict step.

3.3 Geostatistical Model

Binomial count data are modelled within a Bayesian hierarchical modelling framework using a logit link function and a spatially and temporally explicit hierarchical generalised linear regression model to estimate the point prevalence of LRI in the five regions of Africa. Our model was constructed as follows:

$$C_i | p_i,\ N_i \sim \text{Binomial}(p_i,\ N_i)$$

$$\text{logit}(p_i) = \beta_0 + \boldsymbol{X_i \beta} + \epsilon_{GP_i} + \epsilon_{ctry_i} + \epsilon_i$$

$$\sum \boldsymbol{\beta} = 1$$

$$\epsilon_{ctry_i} \sim \text{N}(0, \sigma_{ctry}^2)$$

$$\epsilon_i \sim \text{N}(0, \sigma_{nug}^2)$$

$$\boldsymbol{\epsilon_{GP}} | \boldsymbol{\Sigma}_{\text{space}},\ \boldsymbol{\Sigma}_{time} \sim \text{GP}(0,\ \boldsymbol{\Sigma}_{\text{space}} \otimes \boldsymbol{\Sigma}_{time})$$

$$\boldsymbol{\Sigma}_{\text{space}} = \frac{2^{1-\nu}}{\tau \times \Gamma(\nu)} \times (\kappa \boldsymbol{D})^{\nu} \times \text{K}_{\nu}(\kappa \boldsymbol{D})$$

$$\Sigma_{time_{j,k}} = \rho^{|\,t_k - t_j|}.$$

For each region, we modelled the number of children at location-time i, among a sample size, N_i, who were afflicted with LRI as binomial count data, C_i. The counts, C_i, probabilities, p_i, predictions from the four child models $\boldsymbol{X_i}$, and residual terms ϵ_* are all indexed at a space-time coordinate. The term p_i represents both the annual prevalence and the annual probability that an individual child will be afflicted with an LRI given the child resides at that particular location. The logit of annual prevalence, logit(p_i), was modelled as a linear combination of the

four child models, $\boldsymbol{X_i}$; a correlated spatio-temporal error term, ϵ_{GP_i}; and an independent error term, ϵ_i. Coefficients, $\boldsymbol{\beta}$, on the child models represent their respective predictive weighting in the mean logit link and are constrained to sum to one in an attempt to produce an unbiased estimate as a weighted average of the predicted values from each of the children stackers. ϵ_{ctry_i} is a country random effect, and ϵ_i, is an independent error term. ϵ_{GP}, is modelled as a three-dimensional Gaussian process in space-time centred at zero and with a covariance matrix constructed from a Kroenecker product of spatial and temporal covariance kernels. The spatial covariance, Σ_{space}, is modelled using an isotropic and stationary Matérn function[6], and temporal covariance, Σ_{time}, as an autoregressive order 1 (AR1) function represented in the model with four equally spaced knots.

This approach leveraged the data's residual correlation structure to more accurately predict prevalence estimates for locations with no data, while also propagating the dependence in the data through to uncertainty estimates[7]. The posterior distributions were fit using computationally efficient and accurate approximations in R-INLA[8,9] (integrated nested Laplace approximation) with the stochastic partial differential equations (SPDE)[10] approximation to the Gaussian process residuals.

3.4 Priors

The following priors were used:

- $\beta_0 \sim N(\mu = 0, \sigma^2 = 1000)$,
- $\boldsymbol{\beta} \sim N(\boldsymbol{\mu}, \Sigma\ )$,
    - $\boldsymbol{\mu} = \left(\frac{\mathbf{1}}{\mathbf{4}}, \frac{\mathbf{1}}{\mathbf{4}}, \frac{\mathbf{1}}{\mathbf{4}}, \frac{\mathbf{1}}{\mathbf{4}}\right)'$
    - $\Sigma\ = 1{,}000 * I_{4\,x\,4}$
- $log\left(\frac{1+\rho}{1-\rho}\right) \sim N(\mu = 0, \sigma^2 = 1/0.15)$,
- $\left(\frac{1}{\sigma^2_{ctry}}\right) \sim gamma(\alpha = 1, \gamma = 2)$,
- $\theta_1 = \log(\tau) \sim N(\mu_{\theta_1}, \sigma^2_{\theta_1})$,
- $\theta_2 = \log(\kappa) \sim N(\mu_{\theta_2}, \sigma^2_{\theta_2})$.

We used the uncorrelated multivariate normal priors that INLA automatically determines based on the finite elements mesh for the log-transformed spatial hyperparameters κ and τ. The mean (μ) and variance (σ^2) parameters for the hyperpriors selected by INLA for the meshes in each region can be found in Supplementary Table 4. In our parameterisation we represent α and γ in the *gamma* distribution as rate and shape, respectively.

3.5 Mesh Creation

We constructed the finite elements mesh for the stochastic partial differential equation approximation to the Gaussian process regression using a simplified polygon boundary for each of the regional strata. We set the inner mesh triangle maximum edge length (the mesh size

for areas over land) to be 0.2 decimal degrees, and the buffer maximum edge length (the mesh size for areas over the ocean) to be 5.0 decimal degrees. An example finite elements mesh constructed for eastern sub-Saharan mesh can be found in Supplementary Figure 10.

3.6 Fitted parameters and estimate generation

Fitted parameters and hyperparameters, as well as their 95% CIs are shown by indicator and region in Supplementary Table 5. Spatial hyperparameters (τ and κ) and their uncertainties have been transformed into the more interpretable nominal variance and range parameters. Nominal variance, approximating the variance at any single point, is calculated as $nom.var = 4\pi\kappa^2\tau^2$, and nominal range, approximating the distance before spatial correlation decays by 90%, as $range = \sqrt{8}/\kappa$.

All estimates were generated by taking 1,000 draws from the posterior distribution. For estimates at the pixel level, these draws were used directly to generate estimates and uncertainty. Aggregated estimates, in which estimates at the pixel level were summarised to administrative boundaries, were generated by creating population-weighted averages of pixels within each administrative boundary, for each draw. The 95% CIs around the mean of our estimates (Supplementary Figures 11-13) were generated by taking the 2.5% and 97.5% quantiles of each of the draws, at the pixel or administrative level.

4.0 Post-estimation

4.1 Calibration to the Global Burden of Disease (GBD) 2017

To leverage national-level data included in GBD 2017, but outside the scope of our current geospatial modelling framework and to ensure agreement between these estimates and GBD 2017 national-level estimates, we performed a post-hoc calibration such that the population weighted mean of the 5 × 5 km estimates within a particular country-year recovers the corresponding mean estimate from the GBD[11].

Specifically, for each country-year, we find a value k using the bisection method such that the $\sum \text{invlogit}(\text{logit}(p_i) + k) * pop$ equals the number of prevalent cases reported in GBD, where p_i is the predicted prevalence at a given x/y coordinate, and pop is the population of all children under five at that same coordinate. The summation occurs over all pixels within the selected country-year.

4.2 Conversion of point prevalence to other measures

We converted our calibrated estimates of LRI point prevalence (the output of the model + raking step) to severe incidence and mortality due to LRI by finding translation factors for each country-year derived from the relationship estimated as part of the GBD. As such, this

translation assumes identical relative spatial patterns between LRI prevalence, incidence, and mortality within a particular country-year.

4.3 Fractional pixel aggregation

For ease of computation, we assigned each valid pixel to a particular country based on majority coverage (a standard rasterisation approach). However, we relaxed this rule for first and second level administrative units within a country. For each country, we computed the geometric intersection between each pixel assigned to the country and the country's second administrative units (which are nested within the first level units). We fractionally assigned each pixel to any intersecting second administrative unit by examining the starting area of the pixel and the relative areas of the resulting geometric intersections. This allowed us to get more accurate aggregated estimates and mitigated any rasterisation based oddities—for example, an administrative unit may be assigned as having NA results and population because it could not fulfill the decision rule for pixel assignment (e.g., majority, first coverage, and last coverage).

4.4 Pneumococcal pneumonia attributable fraction

For *Streptococcus pneumoniae* (pneumococcal pneumonia), we calculated the population attributable fraction (PAF) using a vaccine probe design[12,13]. The ratio of vaccine effectiveness against nonspecific pneumonia to pathogen-specific disease represents the fraction of pneumonia cases attributable to a pathogen.

To estimate the PAF for pneumococcal pneumonia, we calculated the ratio of vaccine effectiveness against nonspecific pneumonia to pathogen-specific pneumonia among studies that reported both effectiveness estimates (Equation 1). The PAF from Equation 1 was run through an age-integrating meta-regression tool, accounting for the study type, to obtain age-specific estimates of the PAF for pneumococcal pneumonia in the absence of the vaccine. We included randomized controlled trials (RCTs) and before and after vaccine introduction longitudinal studies. We adjusted the study-level PAF estimate by vaccine coverage and expected vaccine performance to estimate country- and year-specific PAF values. We adjusted the PAF by our estimated *Haemophilus influenzae* type B (Hib) PAF estimate and by vaccine serotype coverage. Because of lack of data describing vaccine efficacy in children younger than 1 month, we did not attribute pneumococcal pneumonia to LRI deaths in this age group.

Equation 2 shows the final equation used to calculate the attributable fraction for pneumococcal pneumonia where we account for the vaccine coverage, serotype specific coverage, vaccine effectiveness, and the attributable fraction for Hib.

1) $$StrepPAF_{Base} = \frac{VE_{Pneumonia} * (1 - PAF_{Hib} * VE_{Hib\ Optimal})}{VE_{Streptococcus} * Cov_{Serotype}}$$

2) $FinalPAF_{Strep} = \frac{StrepPAF_{Base}*(1-Cov_{PCV}*\boldsymbol{Cov_{Serotype}}*VE_{PCV\ Optimal})}{(1-\boldsymbol{HibPAF_{Base}}*Cov_{Hib}*VE_{Hib\ Optimal})*\left(1-\frac{StrepPAF_{Base}*Cov_{PCV}*\boldsymbol{Cov_{Serotype}}*VE_{PCV\ Optimal}}{(1-\boldsymbol{HibPAF_{Base}}*Cov_{Hib}*VE_{Hib\ Optimal})}\right)}$

Where $VE_{Pneumonia}$ is the vaccine efficacy against nonspecific pneumonia, $VE_{Streptococcus}$ is the vaccine efficacy against serotype-specific pneumococcal pneumonia, $Cov_{serotype}$ is the serotype-specific vaccine coverage for PCV[14], $VE_{Hib\ Optimal}$ is the Hib effectiveness in the community (0.8)[15], PAF_{Hib} is the final PAF for Hib, Cov_{PCV} is the PCV coverage, Cov_{Hib} is the Hib coverage by country, and $VE_{PCV\ Optimal}$ is the vaccine effectiveness in the community (0.8)[16].

After country- and year-specific PAFs for pneumococcal pneumonia were generated through this process, PAF values were multiplied by overall mortality rates obtained through our geostatistical model to generate the pneumococcal pneumonia mortality rates shown in Figure 4.

5.0 Model Validation

5.1 In-sample Validation

To explore residual error over space and time, absolute error (data minus predicted posterior mean estimates at the corresponding pixels) were produced for each modelled region (Supplementary Figure 14).

5.2 Out-of-Sample Validation

We constructed two holdout scenarios to examine the predictive validity of our modelling strategy using spatially stratified five-fold out-of-sample cross-validation.

Scenario 1: We created the validation folds by assigning data temporally proportional to data density and spatially using a modified quad-tree tree algorithm, stratified by modelling region. The algorithm recursively partitions two-dimensional space, alternating between horizontal and vertical splits on the weighted data sample size medians, until the data contained within each spatial partition are of a similar sample size. The depth of recursive partitioning is governed by the target sample size (400) within a partition and the minimum number of survey clusters or pseudo-points allowed within each spatial partition (in this case, a minimum of 125 was used). These spatial partitions were then allocated to one of five folds for cross-validation. Supplementary Figure 15 shows the spatio-temporal pattern of the folds.

Scenario 2: Folds were created by randomly assigning entire data sources, stratified by region, to one of the five folds.

For each scenario and modelling region, we ran the entire modelling process five times – once per fold in addition to the full in-sample runs described above—generating a complete set of out-of-sample predictions. Using these out-of-sample predictions, we then calculated mean error (ME, or bias), root-mean-squared-error (RMSE, which summarises total variance), and

95% coverage of our predictive intervals (the proportion of observed out-of-sample data that falls within our predicted 95% CIs) aggregated to the spatial holdout level (quad-tree leaf for scenario 1 and data source (often a country) for scenario 2). Supplementary Figure 15 shows out-of-sample prediction vs. observed data for the scenario 1 holds outs while Supplementary Figure 16 refers to scenario 2 hold out scheme. Similarly, Supplementary Tables 6-7 summarise in-sample and out-of-sample statistics for scenario 1 and 2, respectively.

5.3 Variations of model specification

We conducted two tests of our model specification to qualitatively assess the performance vs. parsimony tradeoff. The first test examined the performance of our modelling framework under a scenario 2 (see Section 5.2) type hold scheme when we used one BRT child model vs. two. Supplementary Figure 17 displays the results of these tests. While both model specifications featured similar out-of-sample RMSE and coverage estimates, the bias on the two BRT specification was less than $1/3^{rd}$ than the one BRT model. As such, we utilised the two BRT model specification.

The second test we conducted examined how four additional different permutations and simplifications of the modelling strategy affected out-of-sample results under the two difference hold out scheme scenarios described in section 5.2. The additional permutations include: (1) A simple linear model—without stacking or the Gaussian process regression (GPR), (2) A GPR model only, (3) A model with stacking but no GPR, and (4) a model with no stacking, but including the GPR. Supplementary Figure 19 provides a visualisation of the out-of-sample statistics of the five permutations by holdout scheme scenario. In general, the stacking only model (number 3) and the main model (Stacking + GP, see sections S2.0 and S3.0) tend to perform similarly and consistently feature the lowest RMSE. Under the conditions of scenario 2 (holding out entire surveys), these two models also have the lowest absolute bias. While some of the other permutations feature better out-of-sample 95% coverage statistics, most of the permutations are in the same general range (93%–97%).

5.4 INLA prior sensitivity analysis

In order to assess the sensitivity of our estimates to changes in INLA prior specification, we compared results generated by our base model to those generated with models using two alternate sets of INLA priors. Supplementary Table 9 details these base and alternate prior specifications. We compared results generated under each alternate prior scenario to those obtained using the base model by calculating the correlation coefficient between prevalence means at the first administrative subdivision level (Supplementary Tables 10–13). Results from both alternate prior specifications were highly correlated with those generated under the base model ($R > 0.98$).

6.0 Supplemental Results

6.1 Prevalence, incidence, and mortality due to LRI

Supplementary Figures 11–13 provide additional visualisation of calibrated estimates for LRI incidence, prevalence, and mortality, respectively.

6.2 Global Action Plan for Pneumonia and Diarrhea (GAPPD) projections

The Global Action Plan for Pneumonia and Diarrhea (GAPPD) has set two main goals for reducing to burden of disease due to pneumonia: (1) the reduction of deaths due to LRI to less than 3 per 1,000 by 2025 and (2) a 75% reduction in severe incidence of LRI relative to a 2010 baseline[17]. To make a coarse assessment of the probability each pixel or first administrative unit will reach each goal, we calculated the annualised rate of change (AROC) for each pixel/unit while weighting more recent trends more heavily. We then used the resulting AROCs to project our estimates for 2017 forwards to 2025 (assuming a constant spatial distribution of population over the period). Finally, we calculated the fraction of projected draws for each pixel or unit that met the GAPPD goals resulting in the probability that a particular area will meet the individual goals. Supplementary Figure 3 shows the calculated AROC of LRI mortality, the probability a particular area met the GAPPD mortality goal in 2010 and 2017, and the projected probability of meeting the mortality goal by 2025. Supplementary Figure 2 visualises the same information, but for the GAPPD's severe incidence goal.

6.3 Concentration of LRI mortality

We conducted a post-hoc evaluation of risk concentration. We used the Getis-Ord G local statistic, which relates each first-level administrative unit and its neighbors to the total set of first-level administrative units and identifies whether a neighborhood is significantly different from the expected value as informed by the total set[18]. In particular, this compares the average risk of a first administrative unit and its direct neighbors against the average risk across all of Africa. Large positive values correspond to areas whose neighborhood average is higher than average (hot-spots), and large negative values correspond to areas whose neighborhood average is lower than average (cold-spots). In the absence of global spatial autocorrelation, these values may be interpreted to assess significance using appropriate Bonferroni corrections for multiple testing. As this application clearly has larger scale spatial autocorrelations (some by design), the local G statistics cannot be used to assess statistical significance of the hot or cold spots. However, as per Ord and Getis[18], a cutoff based on a Bonferroni correction can be useful in our setting for making inferences. We conducted the local G analysis on both the posterior mean risk by first administrative unit as well as using 1,000 draws from the posterior distribution or risk for each year (Supplementary Figures 4–5).

To assess the potential impact of the modelled spatial autocorrelation included in our estimates, we recreated the entire hot-spot analysis using a model run where the spatial correlation

structure of the model had been removed. These results for the posterior mean show identical qualitative results (Supplementary Figures 5–6, respectively).

4.0 Supplementary Figures

Supplementary Figure 1. Geospatial modelling flowchart ... 15

Supplementary Figure 2. Lower respiratory infection incidence annualised rate of change and posterior probability of meeting GAPPD goal for 2025 ... 17

Supplementary Figure 3. Lower respiratory infection mortality annualised rate of change and posterior probability of meeting GAPPD goal for 2017 and 2025 ... 18

Supplementary Figure 4. LRI hot-spot analysis in 2000, 2005, 2010, and 2017 ... 19

Supplementary Figure 5. Draw-level hot-spot sensitivity analysis for LRI in 2000, 2005, 2010, and 2017 ... 20

Supplementary Figure 6. LRI hot-spot analysis in 2000, 2005, 2010, and 2017, alternative specification ... 21

Supplementary Figure 7. Lower respiratory infections data availability by type and country ... 22

Supplementary Figure 8. Covariates ... 23

Supplementary Figure 9. Map of modelling regions ... 23

Supplementary Figure 10. Finite elements mesh ... 25

Supplementary Figure 11. Posterior means and 95% credible intervals for LRI prevalence ... 26

Supplementary Figure 12. Posterior means and 95% credible intervals for LRI incidence ... 27

Supplementary Figure 13. Posterior means and 95% credible intervals for LRI mortality ... 28

Supplementary Figure 14. Plots of lower respiratory infection prevalence absolute error in Africa ... 29

Supplementary Figure 15. Out of sample performance using quad-tree holdouts ... 30

Supplementary Figure 16. Out of sample performance using survey holdouts ... 34

Supplementary Figure 17. Comparison of one vs. two boosted regression tree (BRT) sub-models in stacked generalisation ... 36

Supplementary Figure 18. Out of sample statistics of different model specifications ... 37

Supplementary Figure 19. Quad-tree folds for cross-validation ... 39

Supplementary Figure 20. Seasonal patterns in Lower Respiratory Infections ... 40

Supplementary Figure 1. Geospatial modelling flowchart

The geospatial modelling process consists of four sections. First (in blue), we compiled all available survey data that can be referenced to a coordinate/point (e.g., survey cluster) or small polygon unit and calculated the LRI prevalence at the respective level (S1.0). Data were then adjusted for seasonality and differential recall periods (S1.0). Data matched to polygons were resampled into pseudo points using a k-means clustering algorithm (S2.1). Covariates were subsequently merged to the points and pseudo points via a spatial join (S2.2.2). Second (green), we used the point data and associated covariates and a stacked generalisation ensemble model (S2.2.2). The children models, boosted regression trees, generalised additive models, and elastic net regression were fit using an 5-fold cross-validation process (S2.2.2). The cross-validated predictions from each model then served as the covariate values for the main/parent model (Spatio-temporal GPR model) (S2.2.2–S2.2.7). The predictions from when the child models were fit on all the data (rather than 4/5ths implied by the cross-validation) wered then used to create posterior predictions of LRI prevalence in a 5 × 5 km grid for the years 2000–2017 (S2.2.2). Third (purple and circled orange), we combine the predictions from step 2 and calibrated them such that the population weighted mean LRI prevalence for a particular country-year from our model matched the GBD estimates (S3.3)[1,11,19]. Finally (orange), we aggregated our estimates to first administrative units (S3.3). Using these aggregate estimates and the previously calibrated pixel estimates, we were able to convert prevalence estimates into mortality and incidence estimates and otherwise generate maps of these values (S3.3).

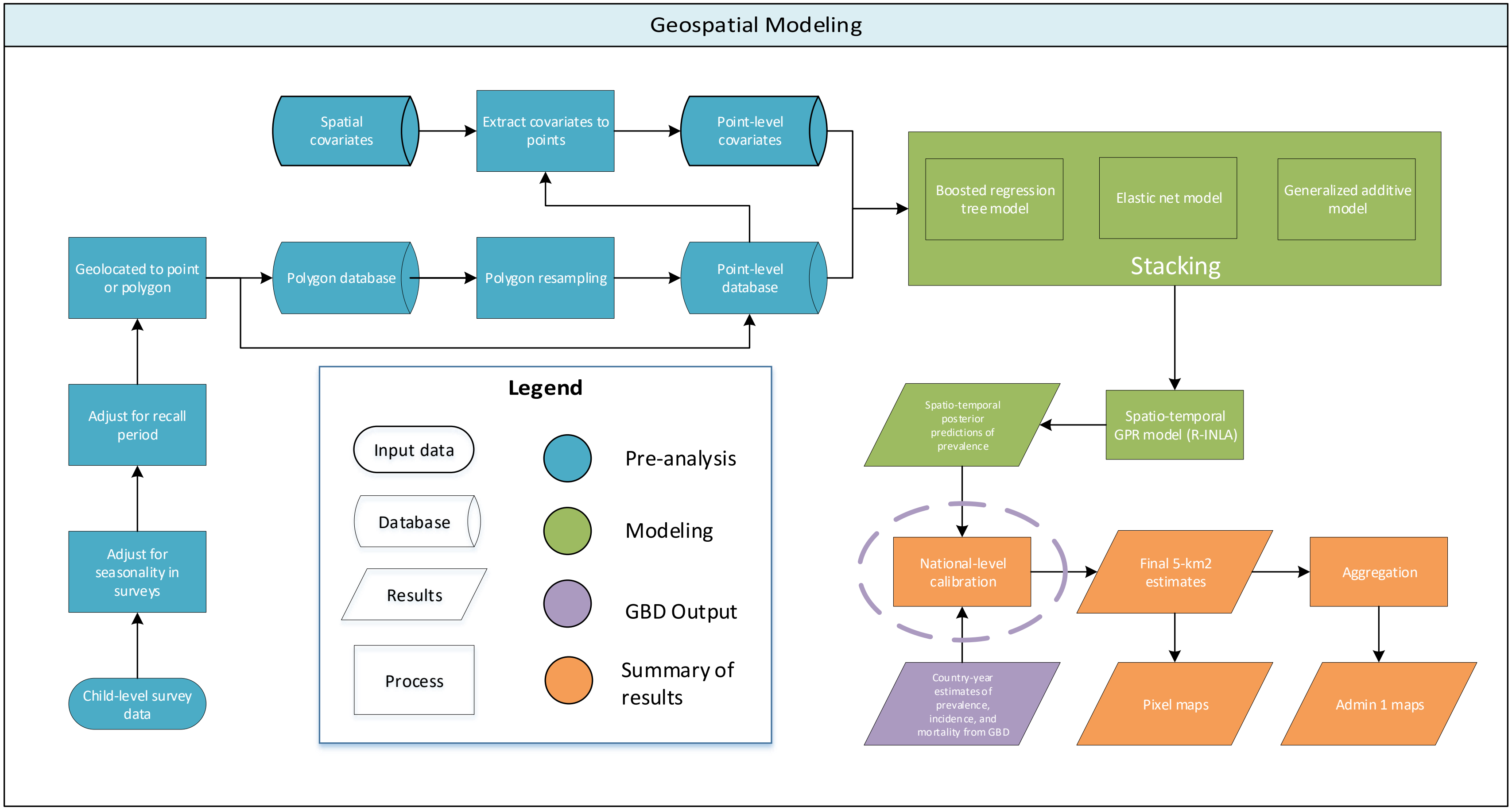
Geospatial Modeling
Spatial covariates
Extract covariates to points
Point-level covariates
Geolocated to point or polygon
Polygon database
Polygon resampling
Point-level database
Boosted regression tree model
Elastic net model
Generalized additive model
Stacking
Adjust for recall period
Adjust for seasonality in surveys
Child-level survey data
Legend
Input data
Database
Results
Process
Pre-analysis
Modeling
GBD Output
Summary of results
Spatio-temporal posterior predictions of prevalence
Spatio-temporal GPR model (R-INLA)
National-level calibration
Final 5-km2 estimates
Aggregation
Country-year estimates of prevalence, incidence, and mortality from GBD
Pixel maps
Admin 1 maps

Supplementary Figure 2. Lower respiratory infection incidence annualised rate of change and posterior probability of meeting GAPPD goal for 2025

(a–b) The estimated annualised decrease in severe LRI incidence from 2000 to 2017. (**c–d)** The posterior probability that the projected 2025 rates of severe LRI incidence reach 75% of the severe incidence rate in 2010. Pixels with fewer than ten people per 1 × 1 km and classified as "barren or sparsely vegetated" are colored in grey.

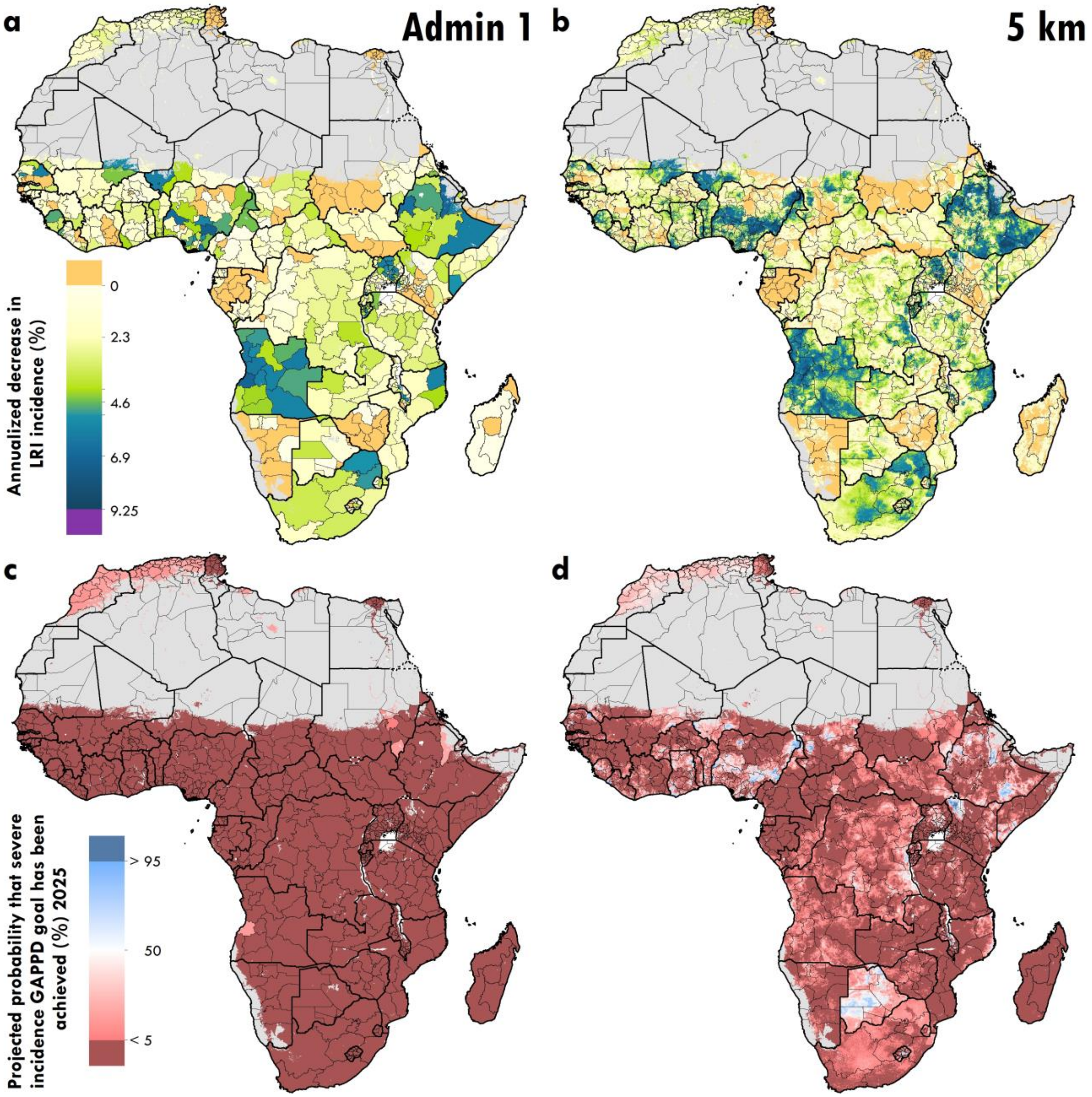

Supplementary Figure 3. Lower respiratory infection mortality annualised rate of change and posterior probability of meeting GAPPD goal for 2017 and 2025

(**a**–**b**) Estimated annualised decrease in LRI mortality from 2000 to 2017. Panels **c** and **d** show the posterior probability that the 2010 rate of mortality attributable to LRI was less than 3 in 1,000. (**e**–**f**) The posterior probability that the 2017 rate of mortality attributable to LRI is less than 3 in 1,000. (**g–h)** The posterior probability that the projected 2025 rates of mortality attributable to LRIs are less than 3 in 1,000 (which is the stated GAPPD goal for 2025). **b**, **d**, **f**, and **h** display the rates at the 5 × 5 km scale at which the model is fit. **a**, **c**, **e**, and **g** display the rates aggregated up to the first administrative subdivision using population weighting. Pixels with fewer than ten people per 1 × 1 km and classified as "barren or sparsely vegetated" are colored in grey.

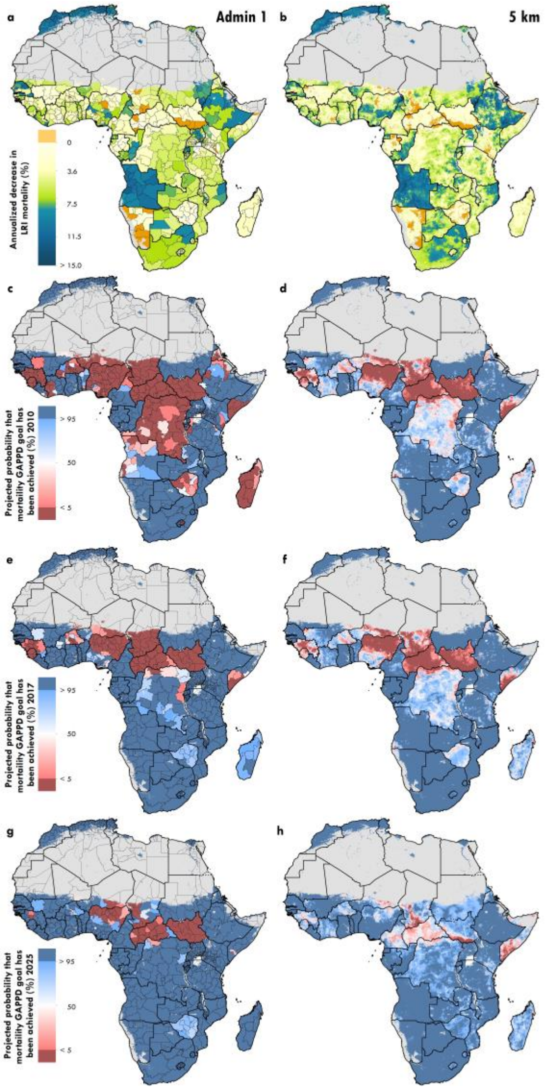

Supplementary Figure 4. LRI hot-spot analysis in 2000, 2005, 2010, and 2017

As in Figure 2e and 2f in the main text, the results of the residual hot-spot analysis are plotted for 2000 (**a**), 2005 (**b**), 2010 (**c**), and 2017 (**d**). There are discontinuities in the color scale at -3.8 and 3.8 to correspond with locations that were identified as cold-spots or hot-spots, respectively.

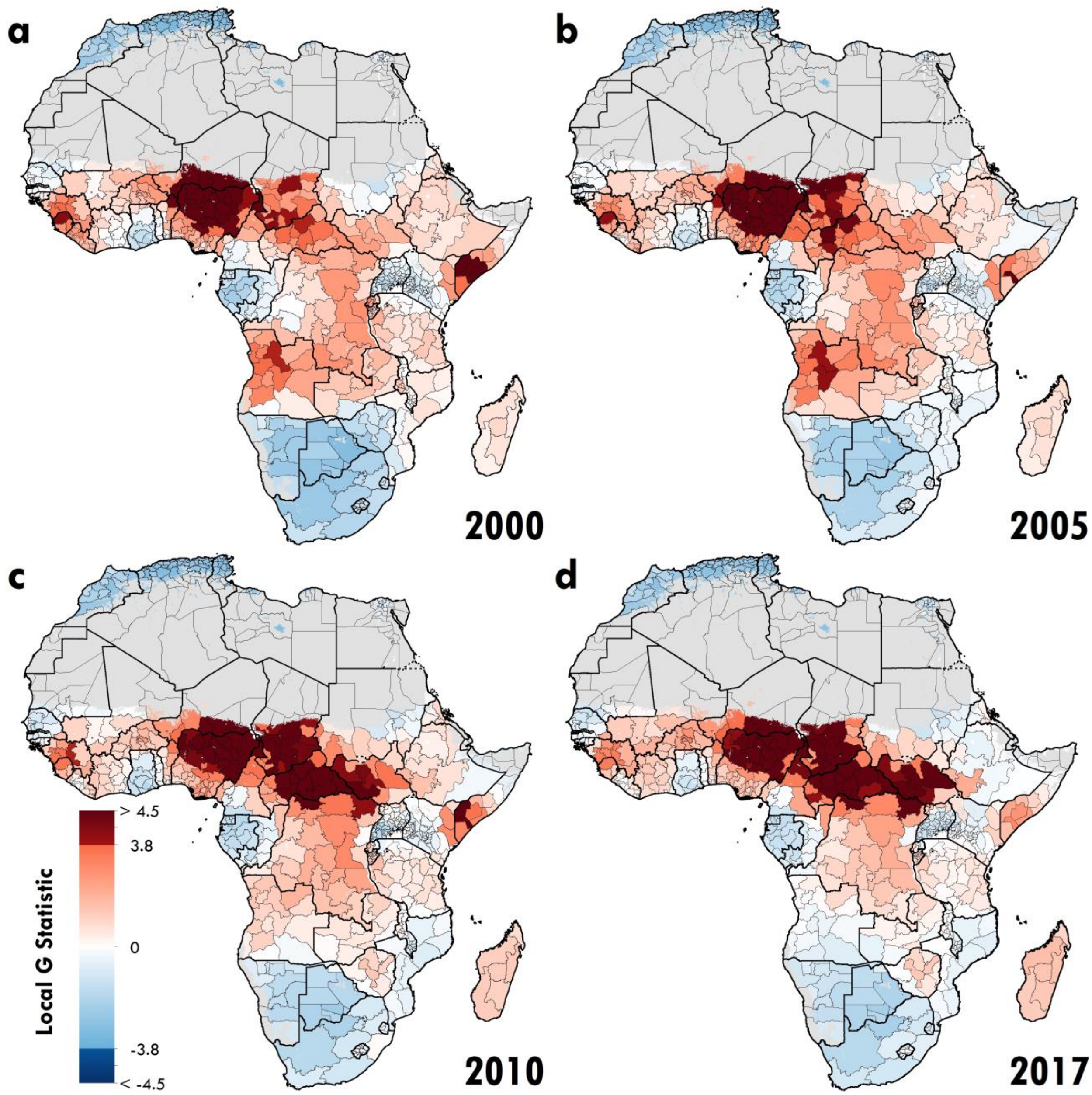

Supplementary Figure 5. Draw-level hot-spot sensitivity analysis for LRI in 2000, 2005, 2010, and 2017

For each posterior draw, a first administrative unit is classified via the G-statistic as a residual hot-spot (or not). Administrative units are colored by the percentage of draws that have been classified as a hot-spot are displayed for 2000 (**a**), 2005 (**b**), 2010 (**c**), and 2017 (**d**).

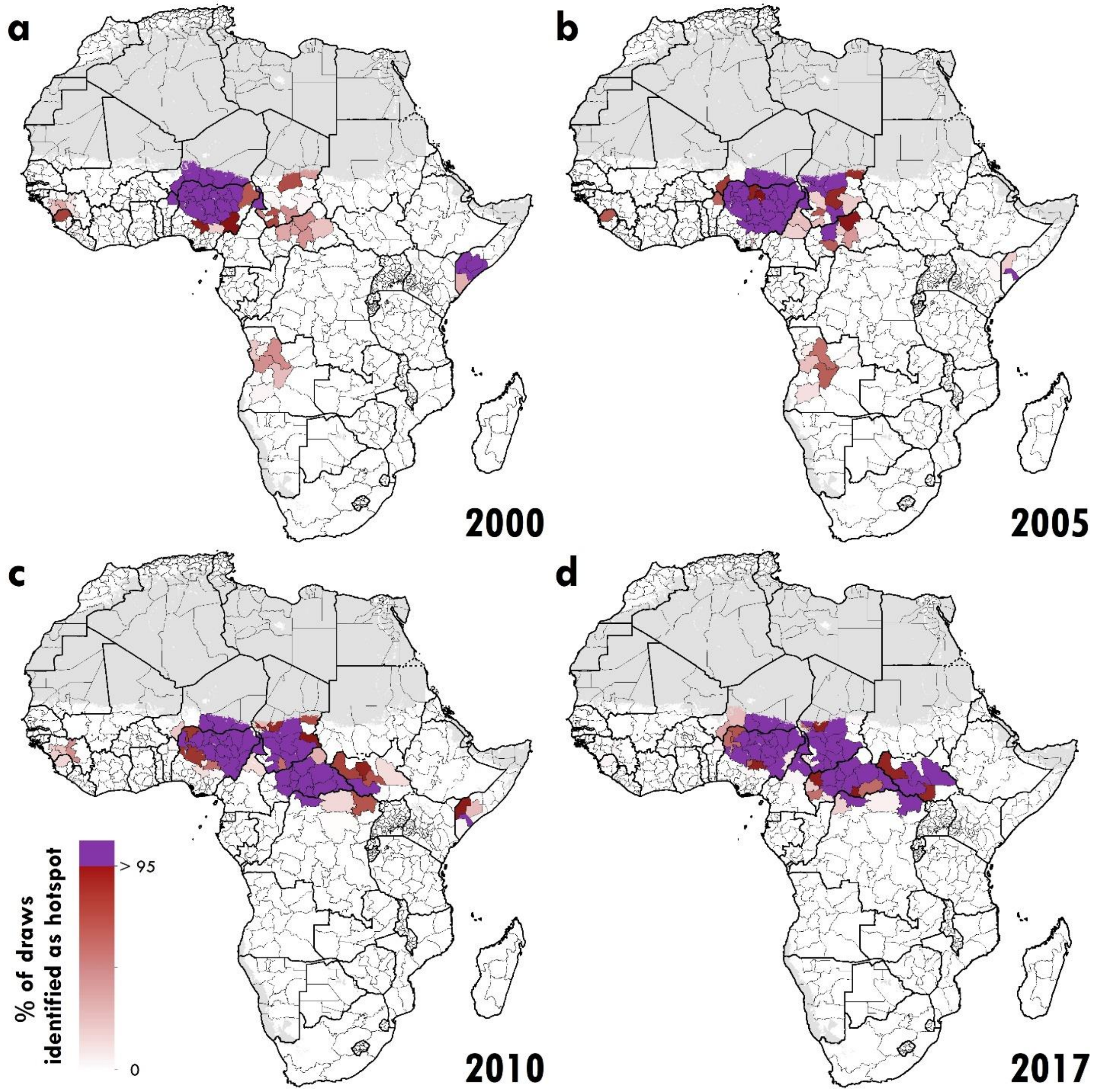

Supplementary Figure 6. LRI hot-spot analysis in 2000, 2005, 2010, and 2017, alternative specification

As in Figure 2e and 2f in the main text, the results of the hot-spot analysis are plotted for 2000 (**a**), 2005 (**b**), 2010 (**c**), and 2017 (**d**). There are discontinuities in the color scale at -3.8 and 3.8 to correspond with locations that were identified as cold-spots or hot-spots, respectively. The results presented here from a model without the spatio-temporal correlation component and show no major differences.

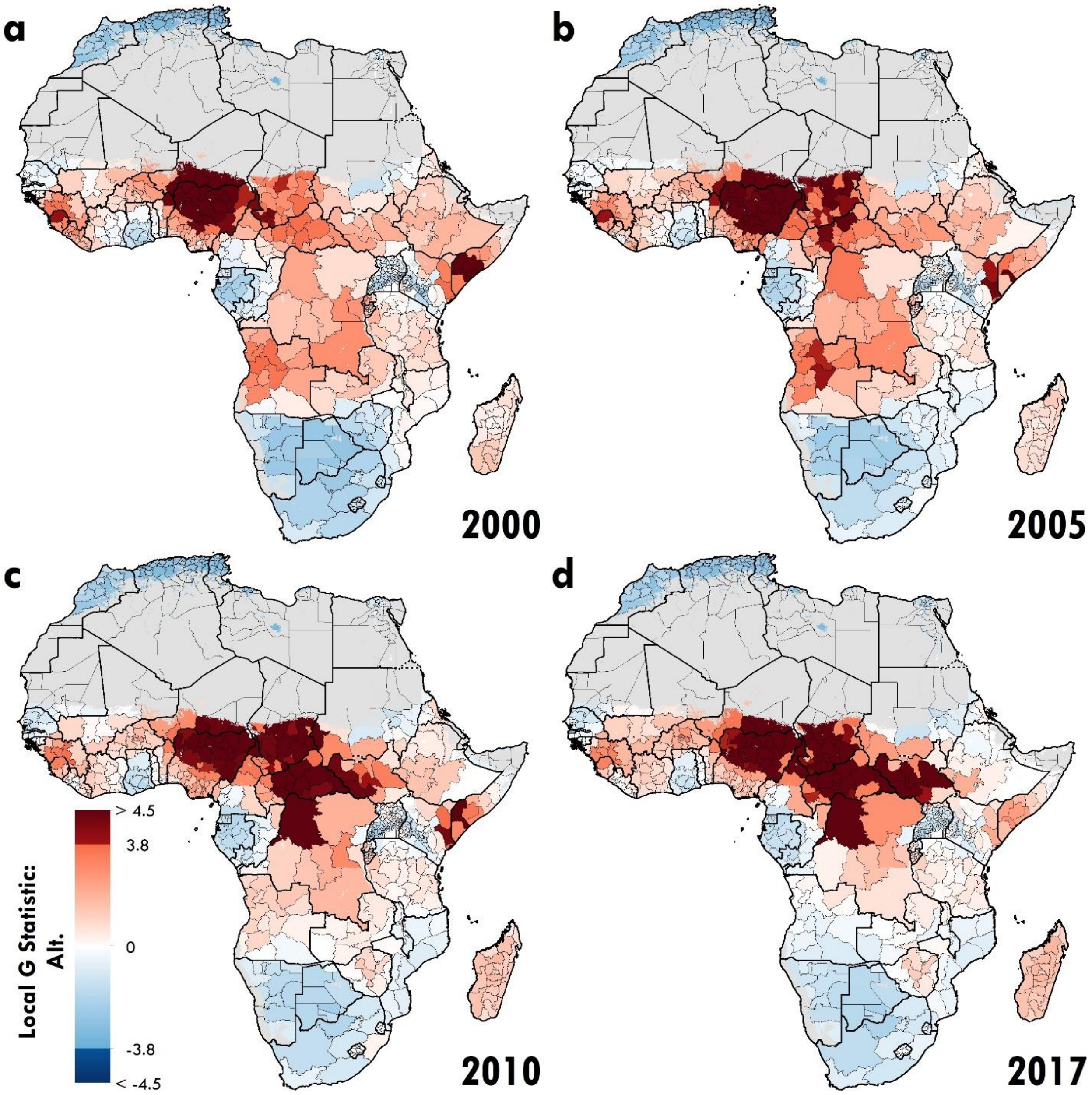

Supplementary Figure 7. Lower respiratory infections data availability by type and country

All data are shown by country and year of survey and are mapped at their corresponding geopositioned coordinate or area. The total number of points and polygons (areal) for each country are plotted by data source, type, and sample size (left side). Sample size represents the number of individual microdata records for each survey. LRI prevalence for the input coordinate or area are mapped (right side). Countries on the map that are coloured fully white indicate no data for the indicated period; countries that are coloured in grey had no data for any year. This database consists of 43,080 survey clusters and 2,639 polygons.

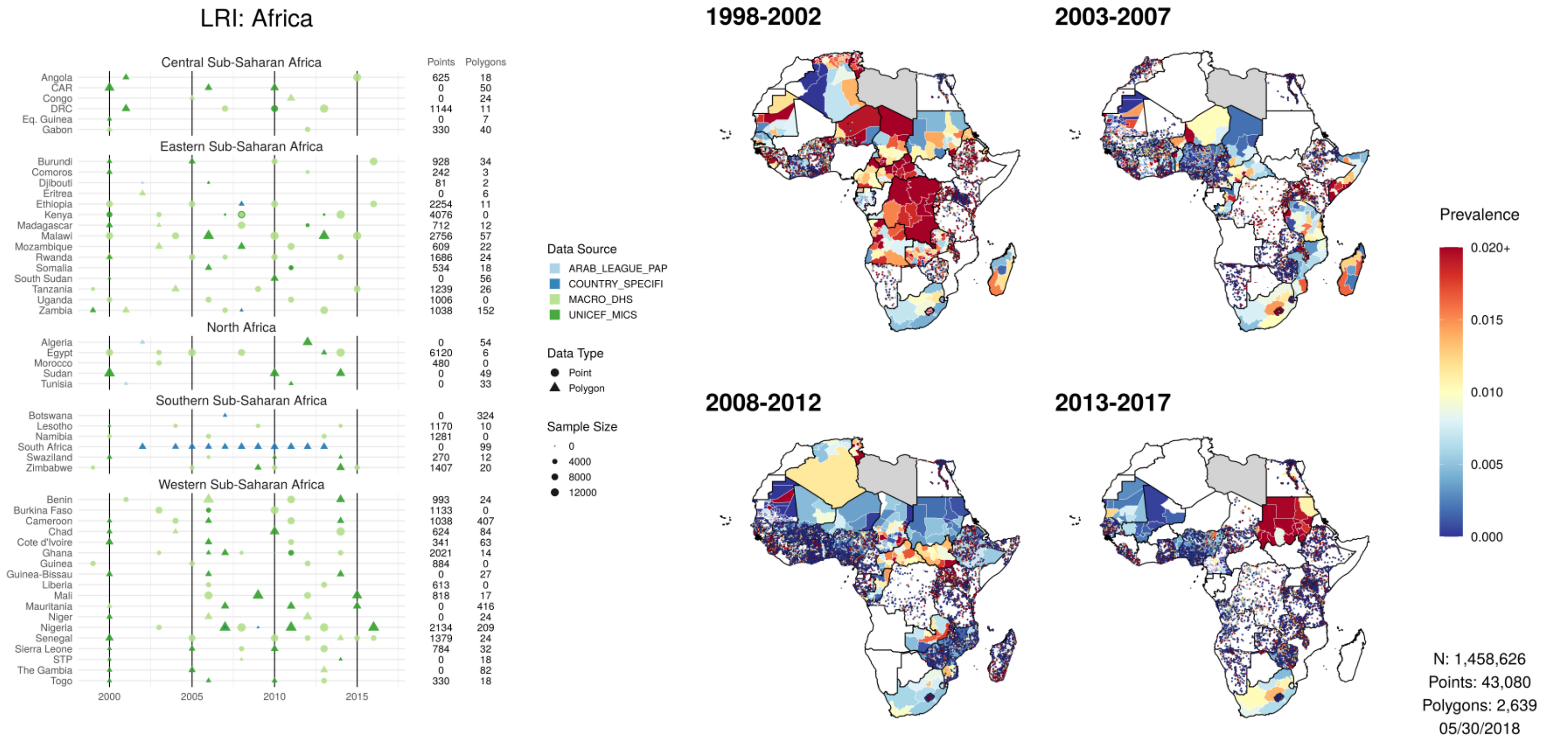

Supplementary Figure 8. Covariates

Twelve covariate raster layers of possible socioeconomic and environmental correlates of LRI prevalence in Africa were used as inputs for the stacking modelling process. Time-varying covariates are presented for the year 2017. For the year of production of non-time-varying covariates, please refer to the individual covariate citation (Supplementary Table 3) for additional detail. Pixels with fewer than ten people per 1 × 1 km and classified as "barren or sparsely vegetated" are colored in grey. PCV coverage is uniformly zero in countries in which the vaccine has not been introduced.

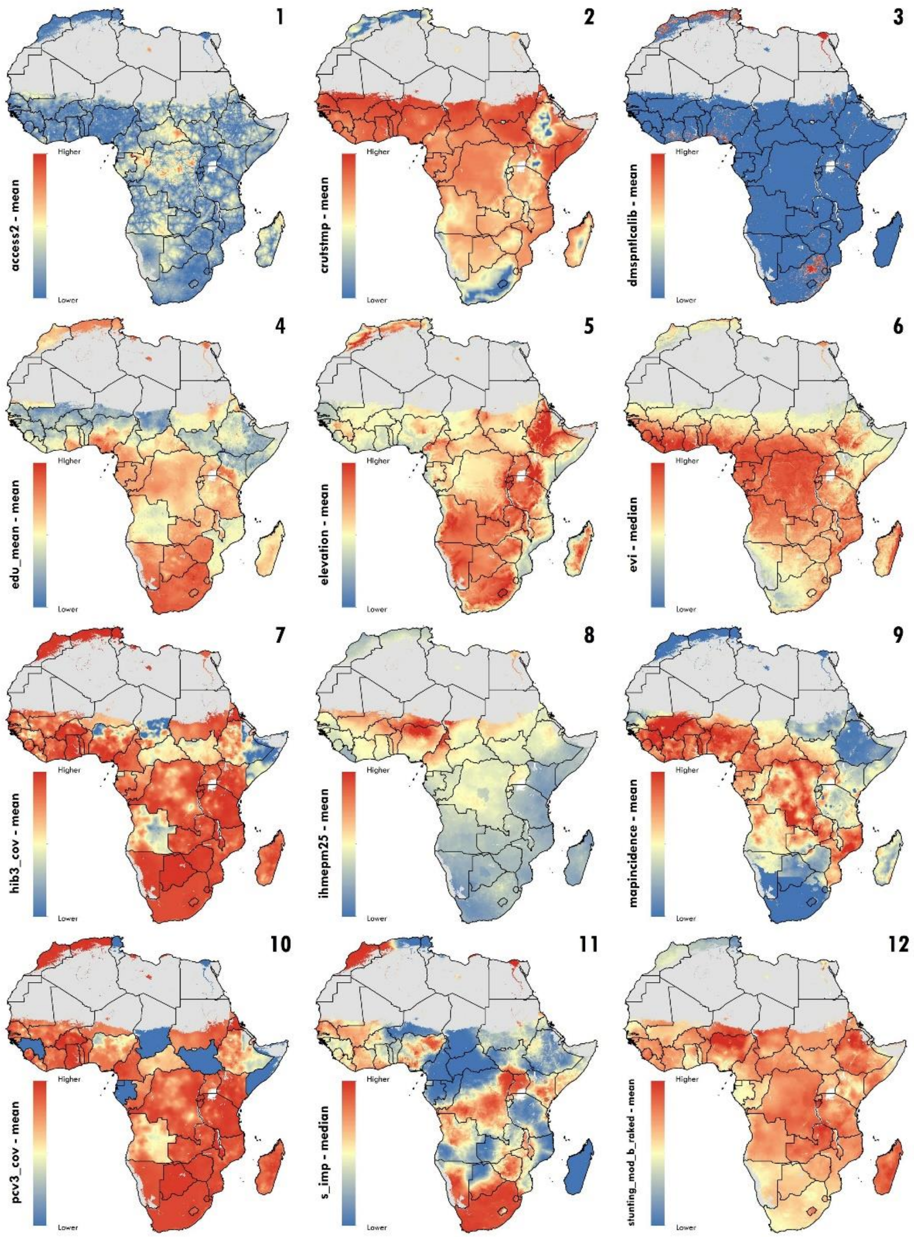

Supplementary Figure 9. Map of modelling regions

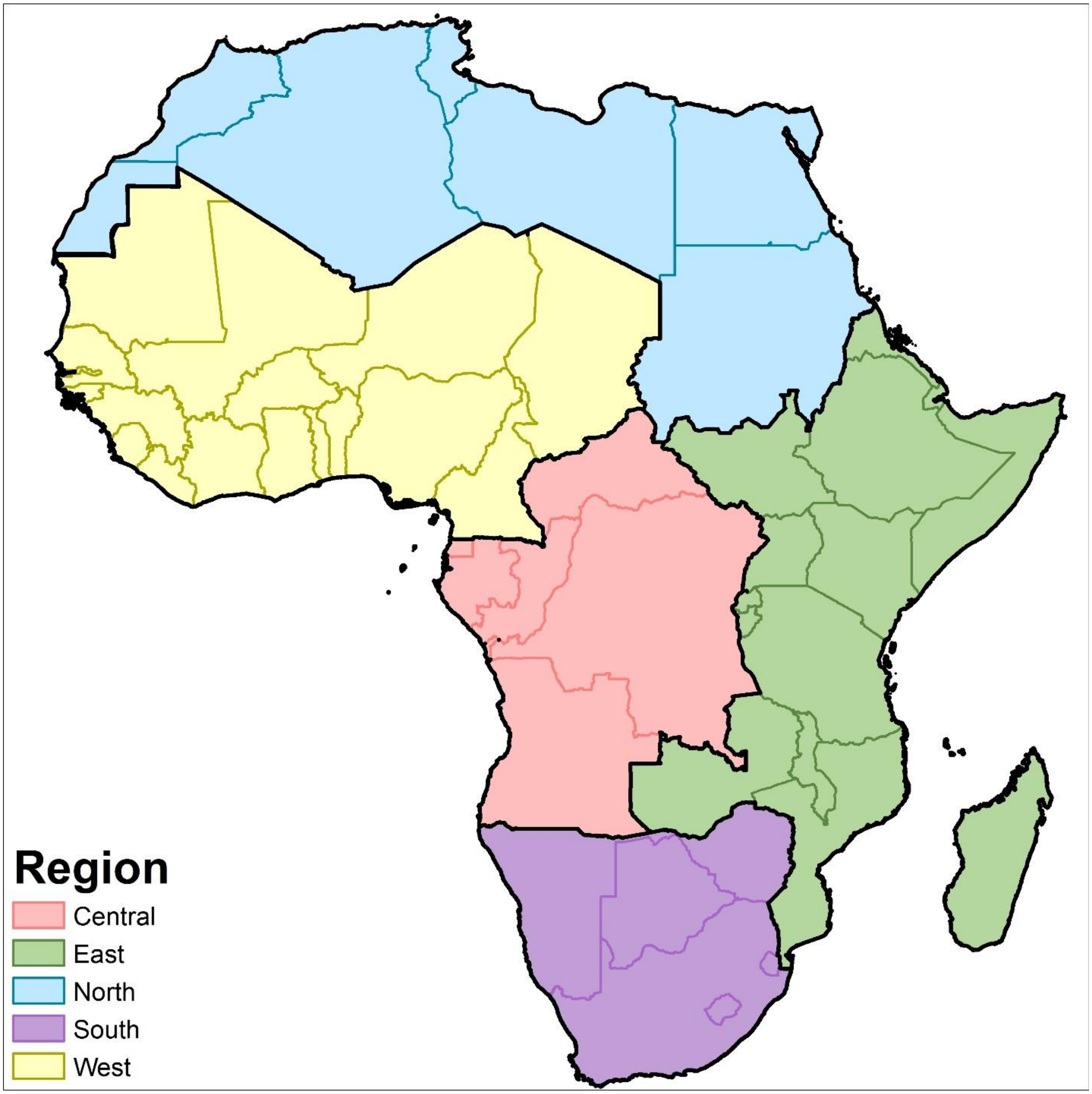

Supplementary Figure 10. Finite elements mesh

The finite elements mesh used to fit the space-time correlated error for the Central Africa region. Both the fine-scale mesh over land in the modelling region and the coarser buffer region mesh are shown. The simplified region polygon used to determine the boundary for the modelling region is shown in blue. The smaller, isolated concentration represents a disconnected area within the same modelling area, such as an island.

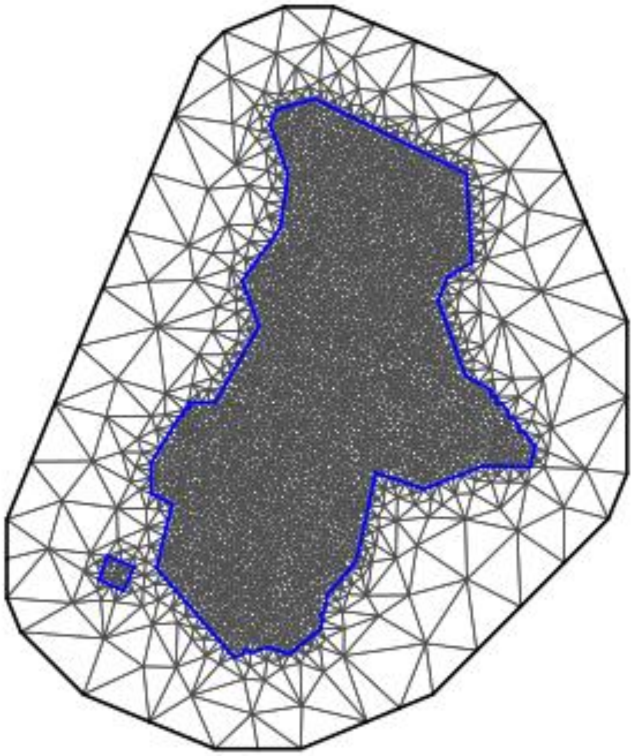

Supplementary Figure 11. Posterior means and 95% credible intervals for LRI prevalence

Pixels with fewer than ten people per 1 × 1 km and classified as "barren or sparsely vegetated" are colored in grey.

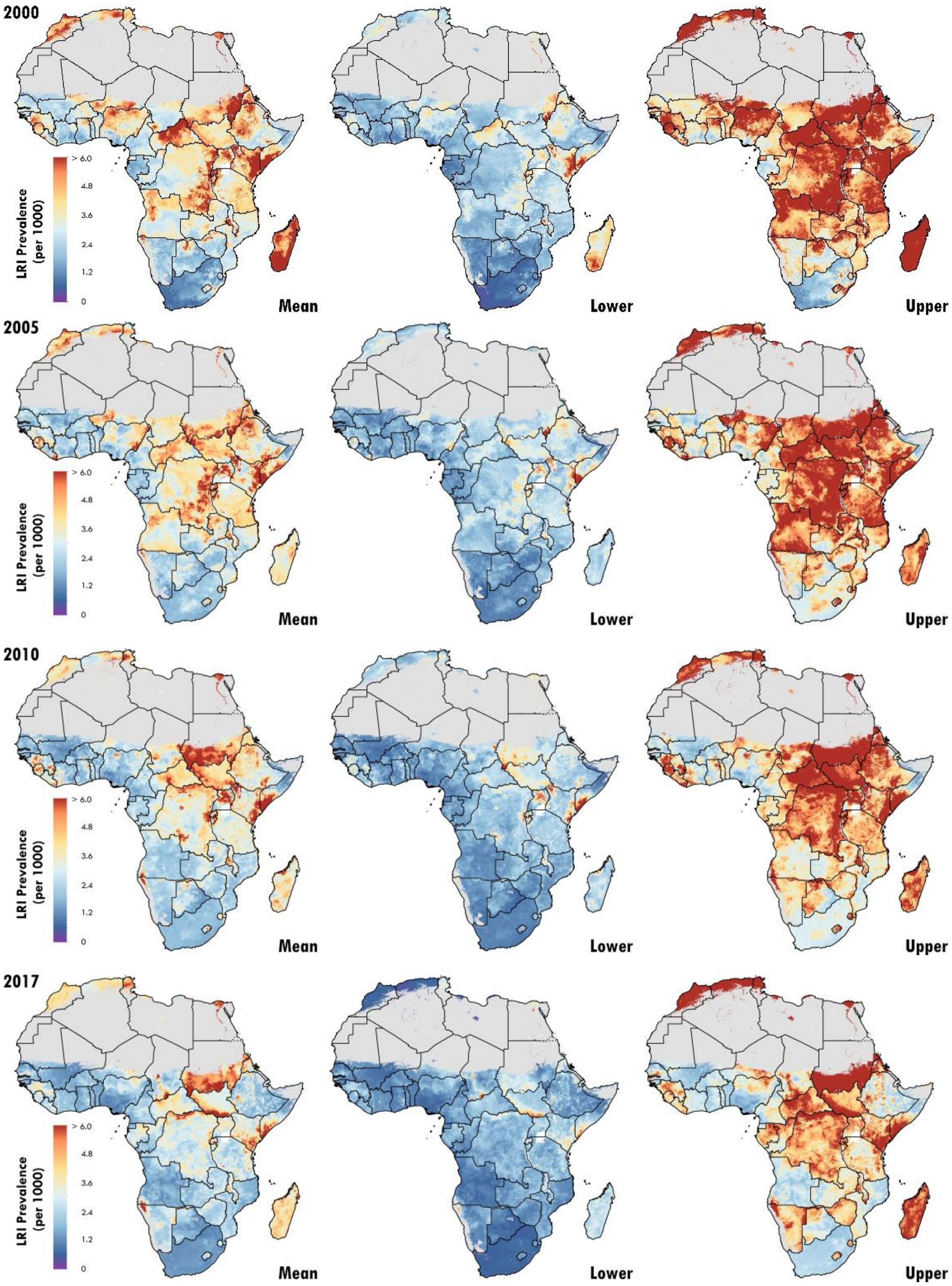

Supplementary Figure 12. Posterior means and 95% credible intervals for LRI incidence

Pixels with fewer than ten people per 1 × 1 km and classified as "barren or sparsely vegetated" are colored in grey.

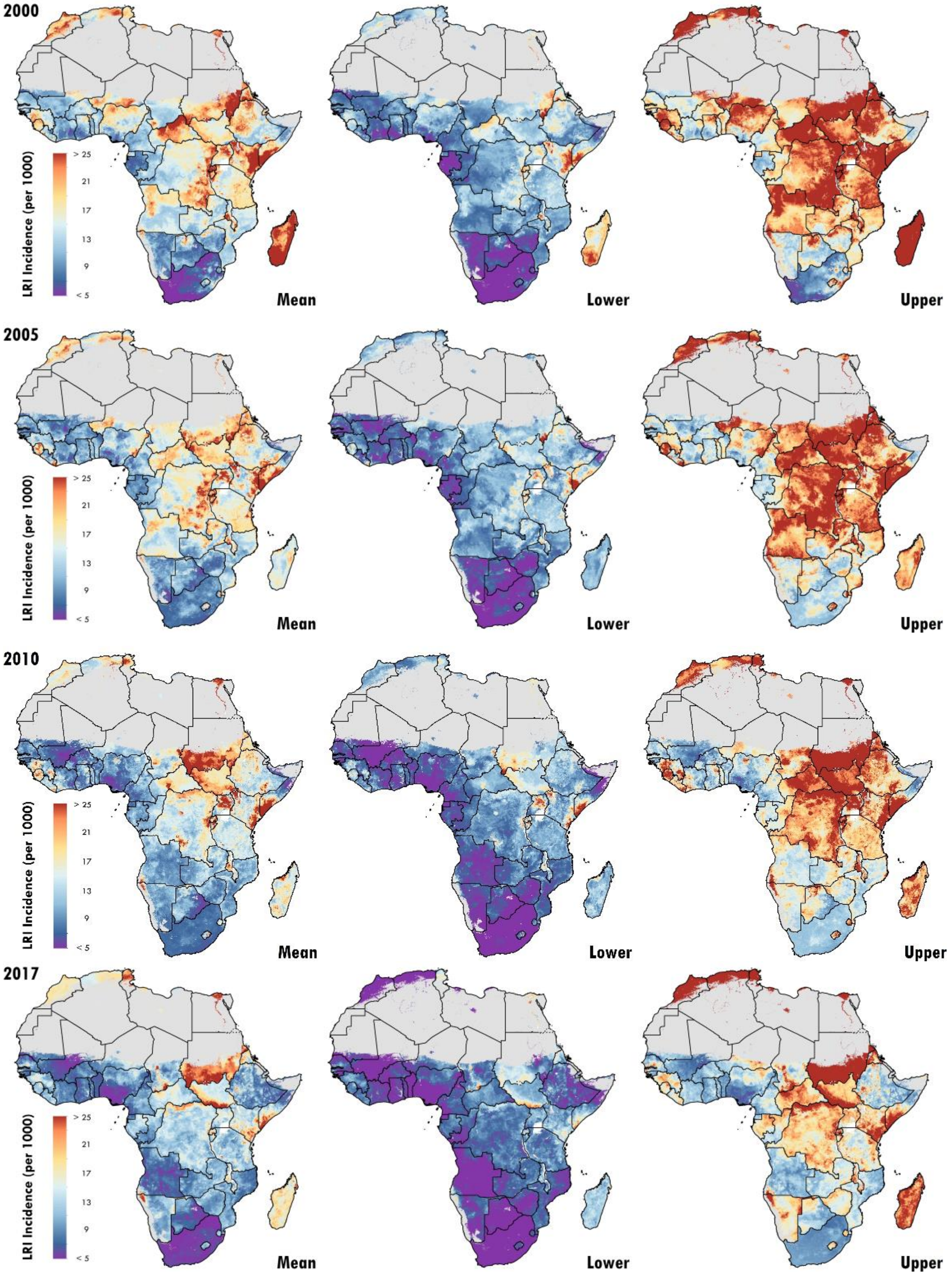

Supplementary Figure 13. Posterior means and 95% credible intervals for LRI mortality

Pixels with fewer than ten people per 1 × 1 km and classified as "barren or sparsely vegetated" are colored in grey.

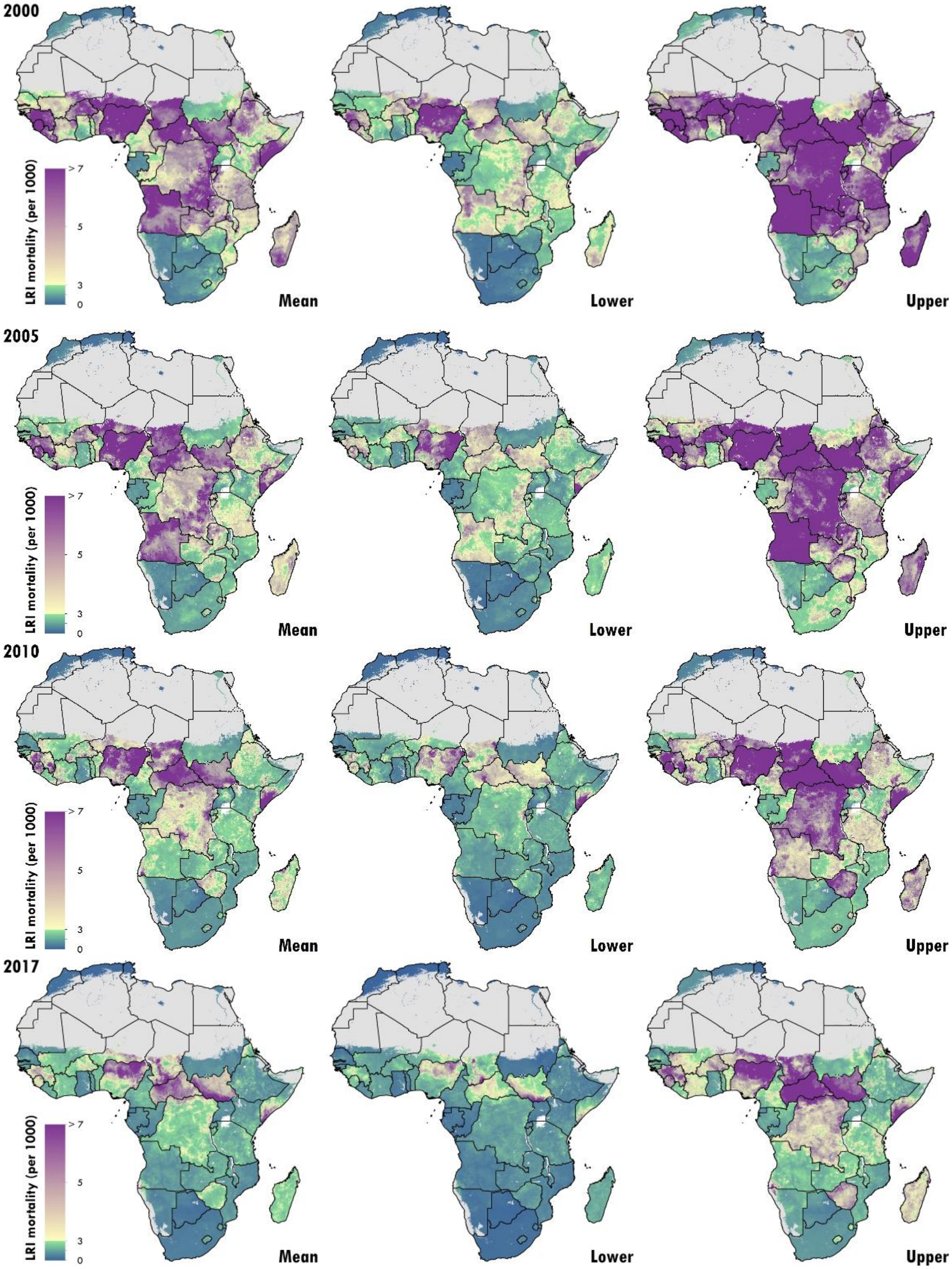

Supplementary Figure 14. Plots of lower respiratory infection prevalence absolute error in Africa

Color indicates magnitude of in-sample error, size of the points represents the sample size of the observed survey cluster or pseudo-cluster, and transparency represents the weight of the survey cluster or pseudo-cluster.

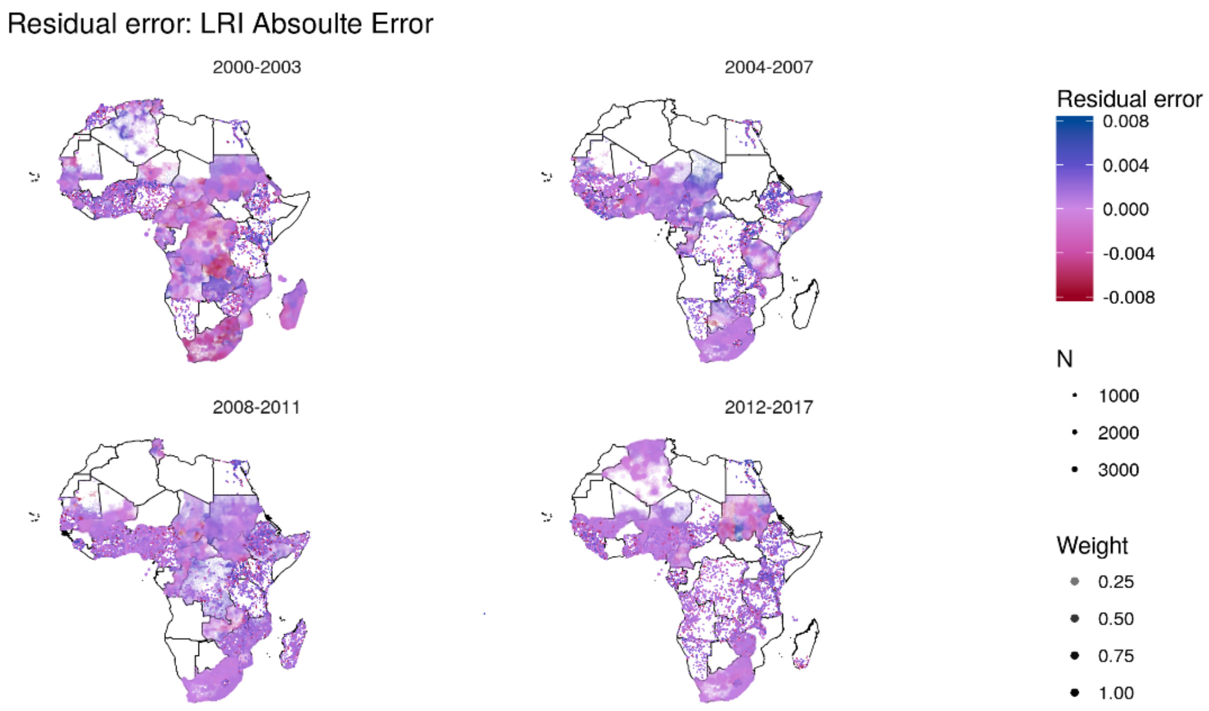

Supplementary Figure 15. Out of sample performance using quad-tree holdouts

Comparison of observed (x-axis) vs. predicted (y-axis) point prevalence of LRI aggregated to the quad-tree spatial holdout (n = 447 spatial holdouts). Weight denotes the sample size of the holdout leaf. Center values show posterior means and error bars indicate 95% credible intervals. (**a**) Points are plotted in 3–4 year groups. (**b**) All years at once. (**c**) Points plotted in 3–4 year groups, zoomed to focus on the bottom left quadrant of the plot. (**d**) All years at once, zoomed to focus on the bottom left quadrant of the plot. The rightmost point in (**b**) corresponds to a holdout containing 3 survey clusters from the 2007 Kenya UNICEF MICS (sample size = 14).

a

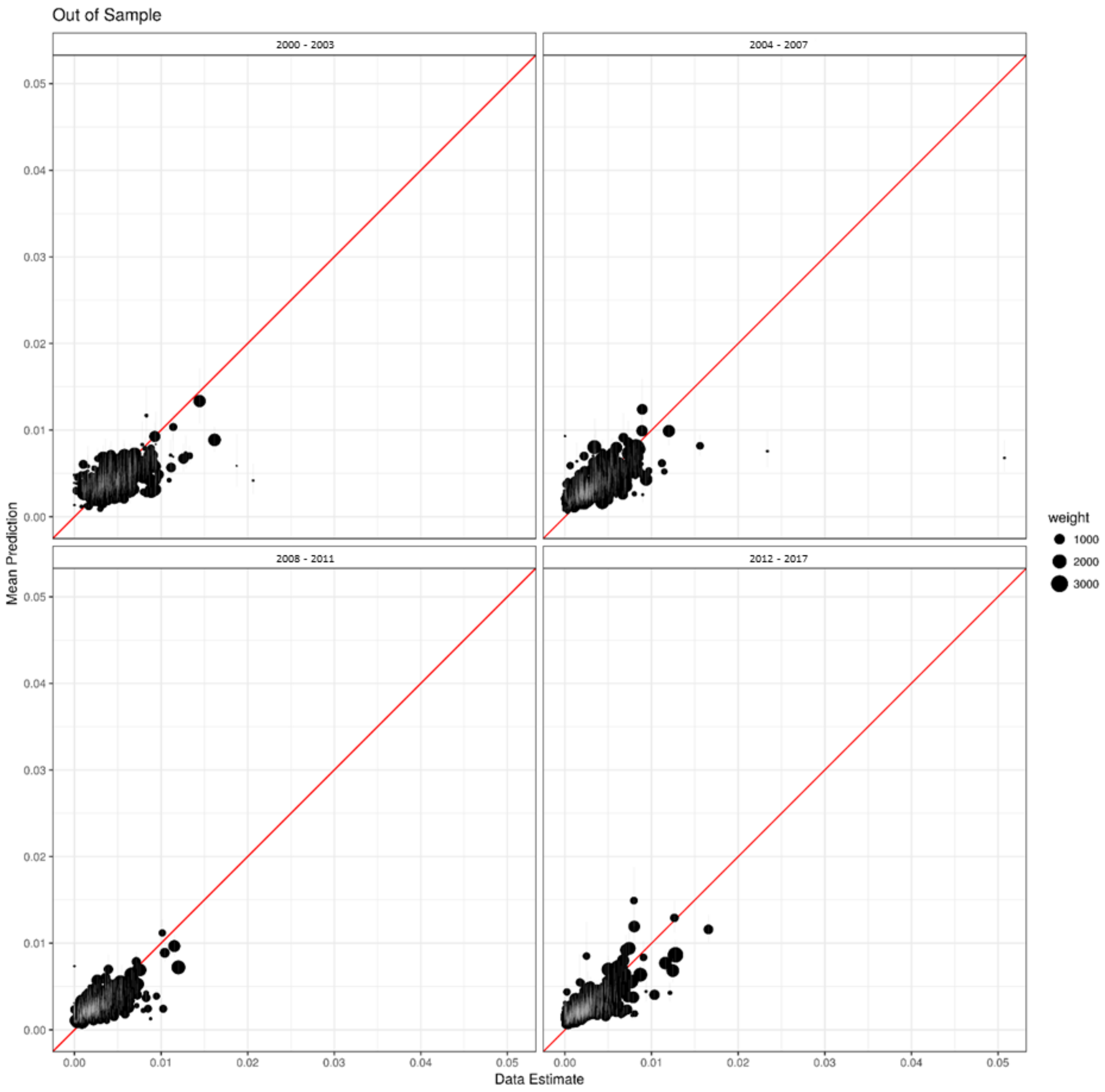

b

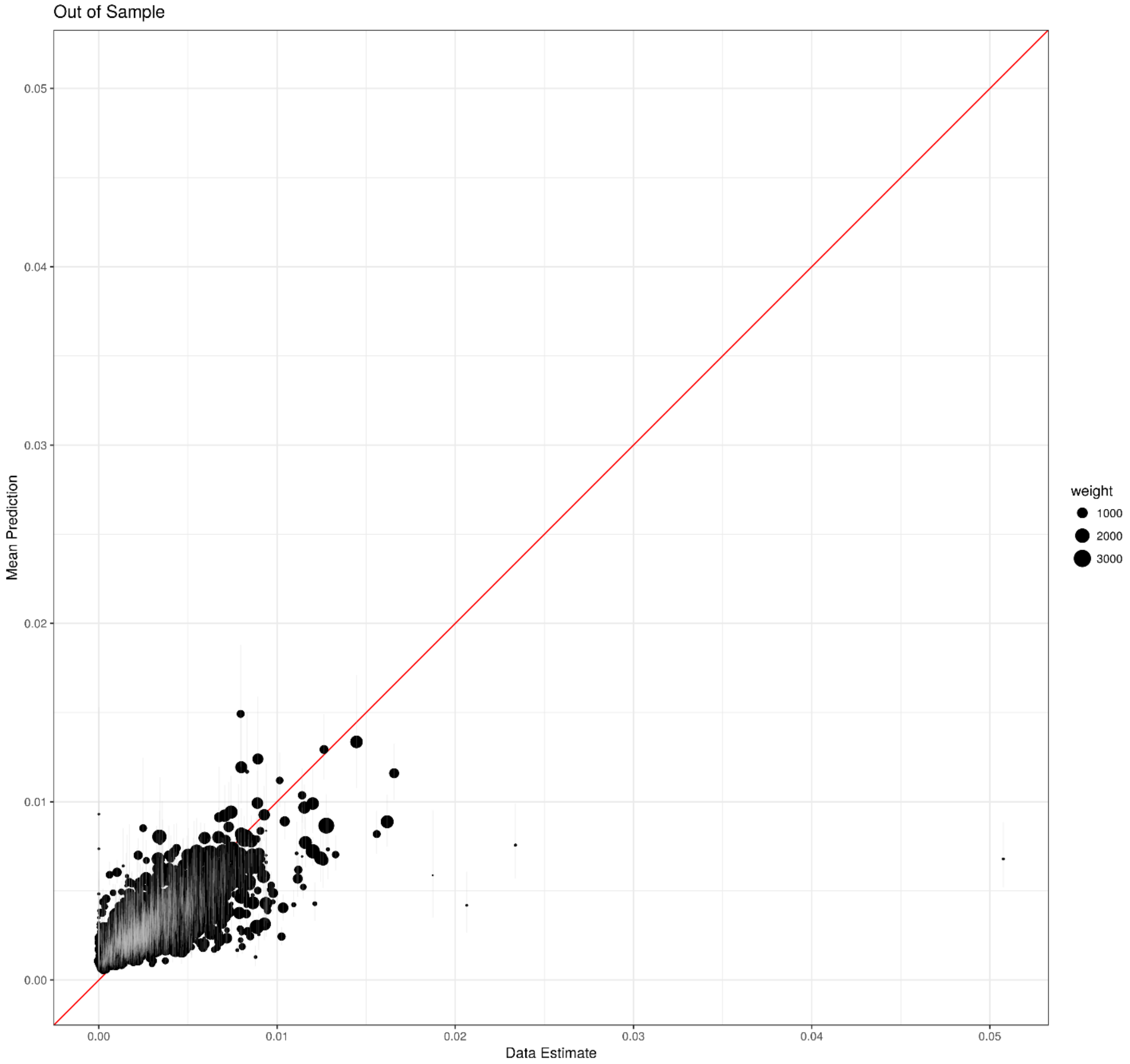
Out of Sample
Mean Prediction
0.05
0.04
0.03
0.02
0.01
0.00
0.00
0.01
0.02
0.03
0.04
0.05
Data Estimate
weight
1000
2000
3000

c

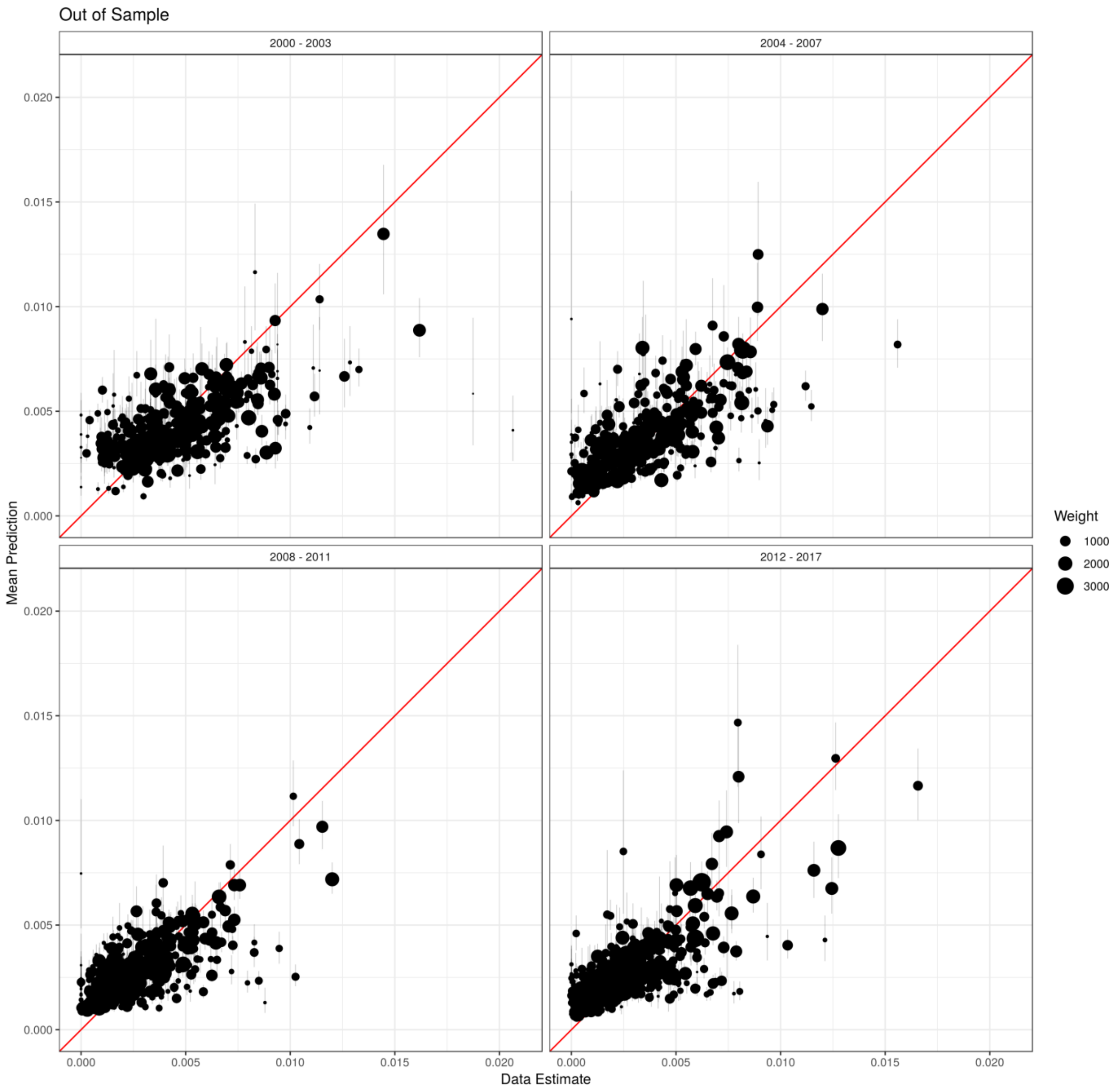
Out of Sample
2000 - 2003
2004 - 2007
2008 - 2011
2012 - 2017
Mean Prediction
Data Estimate
0.000
0.005
0.010
0.015
0.020
Weight
1000
2000
3000

d

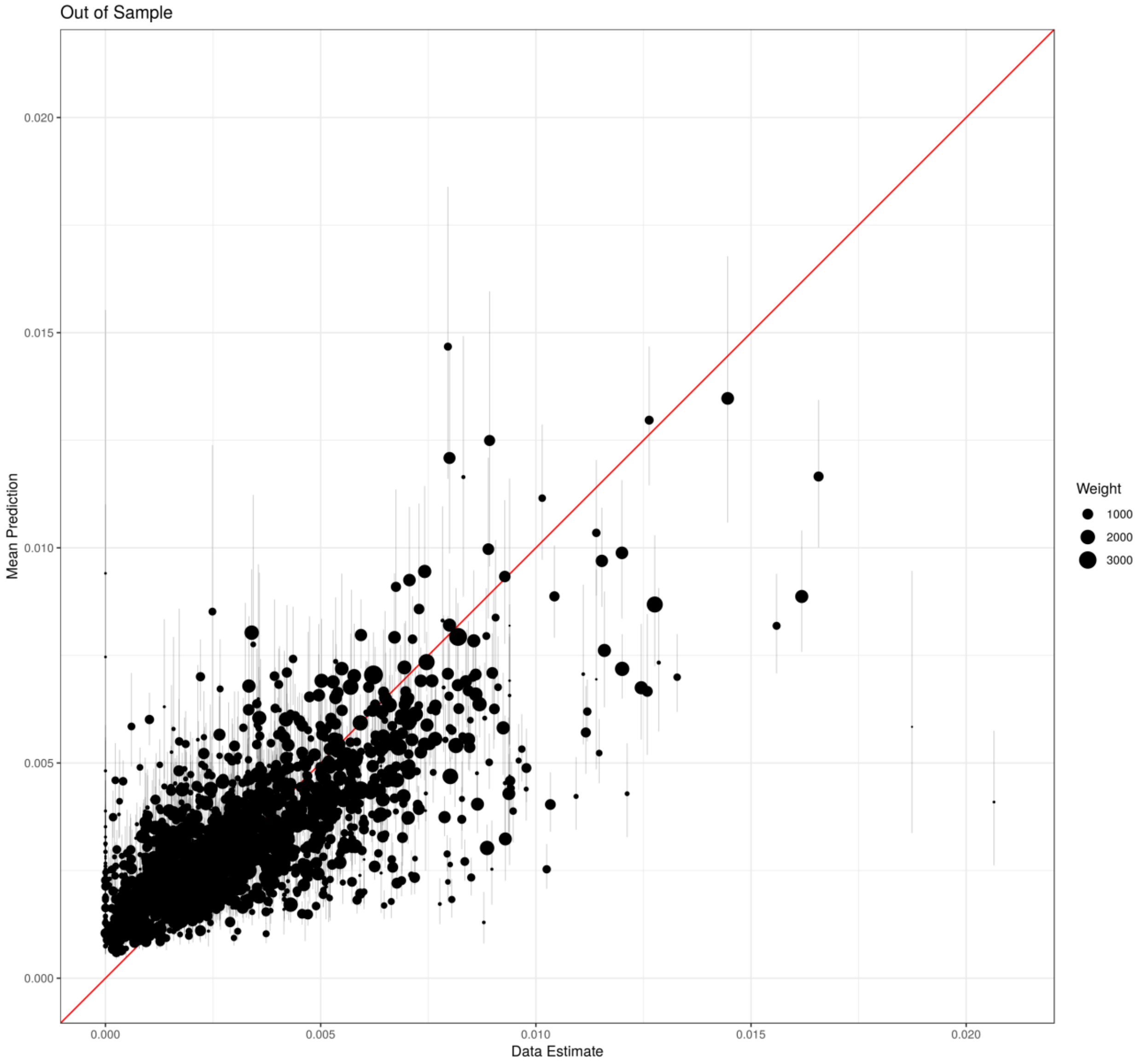

Out of Sample
Mean Prediction
Data Estimate
0.000
0.005
0.010
0.015
0.020
Weight
1000
2000
3000

Supplementary Figure 16. Out of sample performance using survey holdouts

Comparison of observed (x-axis) vs. predicted (y-axis) point prevalence of LRI aggregated to the survey (n = 191 surveys). Weight denotes the sample size of the survey. Center values show posterior means and error bars indicate 95% credible intervals. The surveys grouped into multiple year periods are shown in (**a**) and the entire range is shown in (**b**). The four rightmost points in **(b)** correspond to the following surveys: Lesotho UNICEF MICS (2000), South Africa DHS (2000), Kenya UNICEF MICS (2007), and Sudan UNICEF MICS (2014).

a

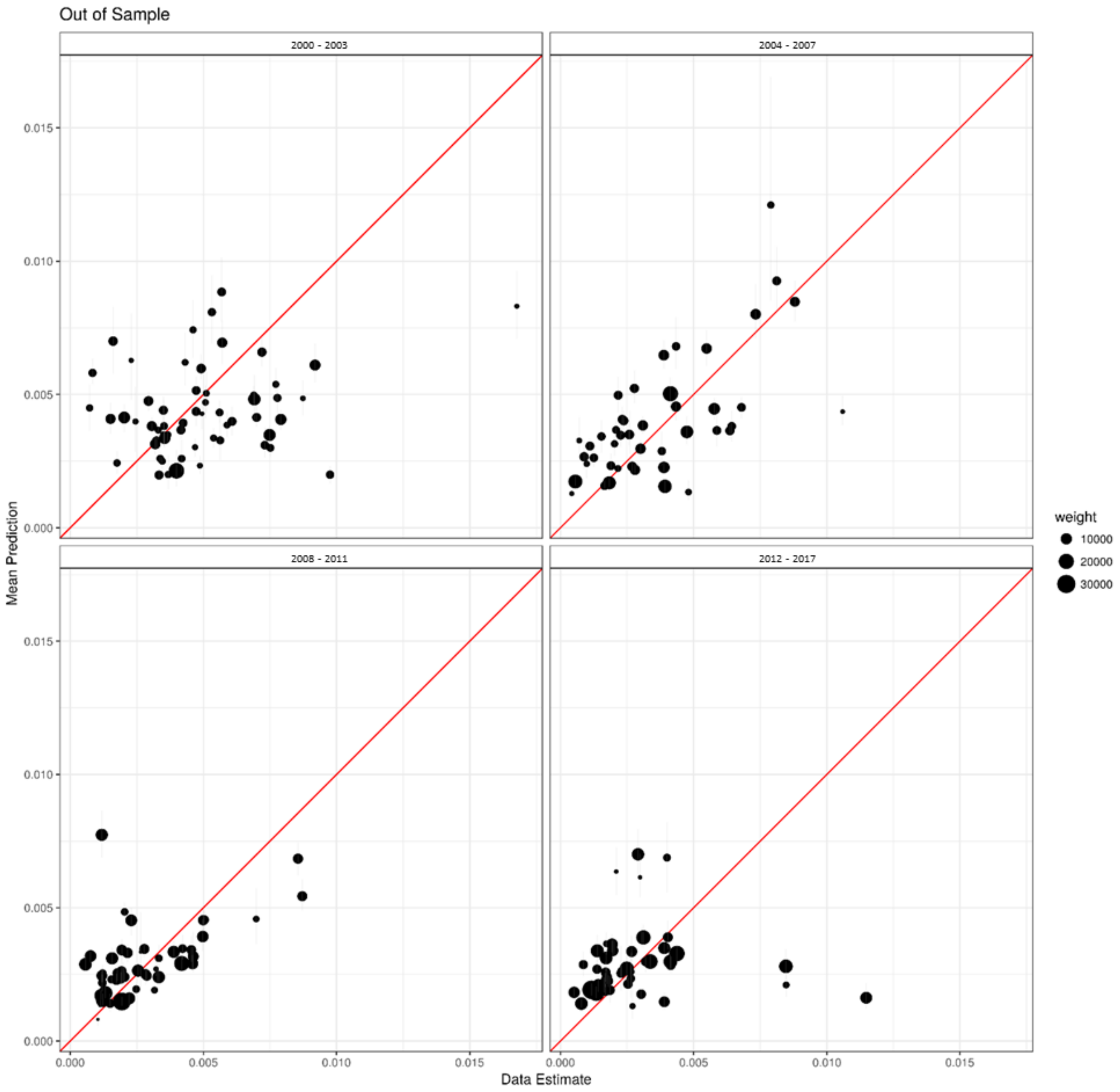

b

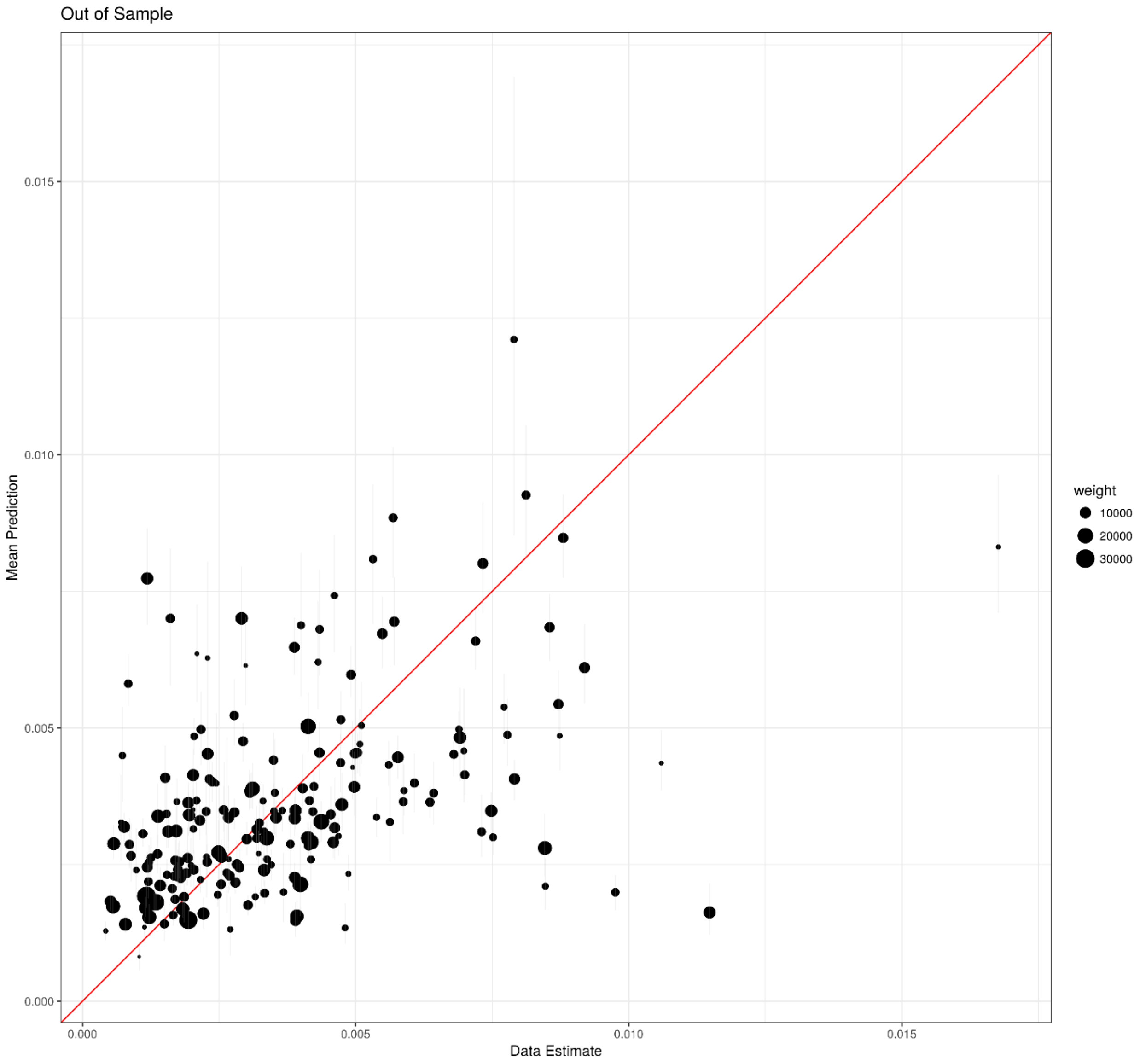

Out of Sample
Mean Prediction
Data Estimate
0.000
0.005
0.010
0.015
0.000
0.005
0.010
0.015
weight
10000
20000
30000

Supplementary Figure 17. Comparison of one vs. two boosted regression tree (BRT) sub-models in stacked generalisation

Coverage vs. RMSE, Bias vs. RMSE, and Coverage vs. Bias are provided for two different specifications of the main stacked generalisation + Gaussian Process Regression model. Validity results were calculated at the survey level and averaged over years. One BRT refers to a model configuration with one optimised BRT sub-model while Two BRTs refers to a model with two optimised BRTs—the BRT sub-model from the one BRT configuration and a second sub-model with similar out of sample performance to the first sub-model but with a minimised correlation (~0.60). BRTs were optimised by three rounds of three-fold cross-validation with data partitioned by survey.

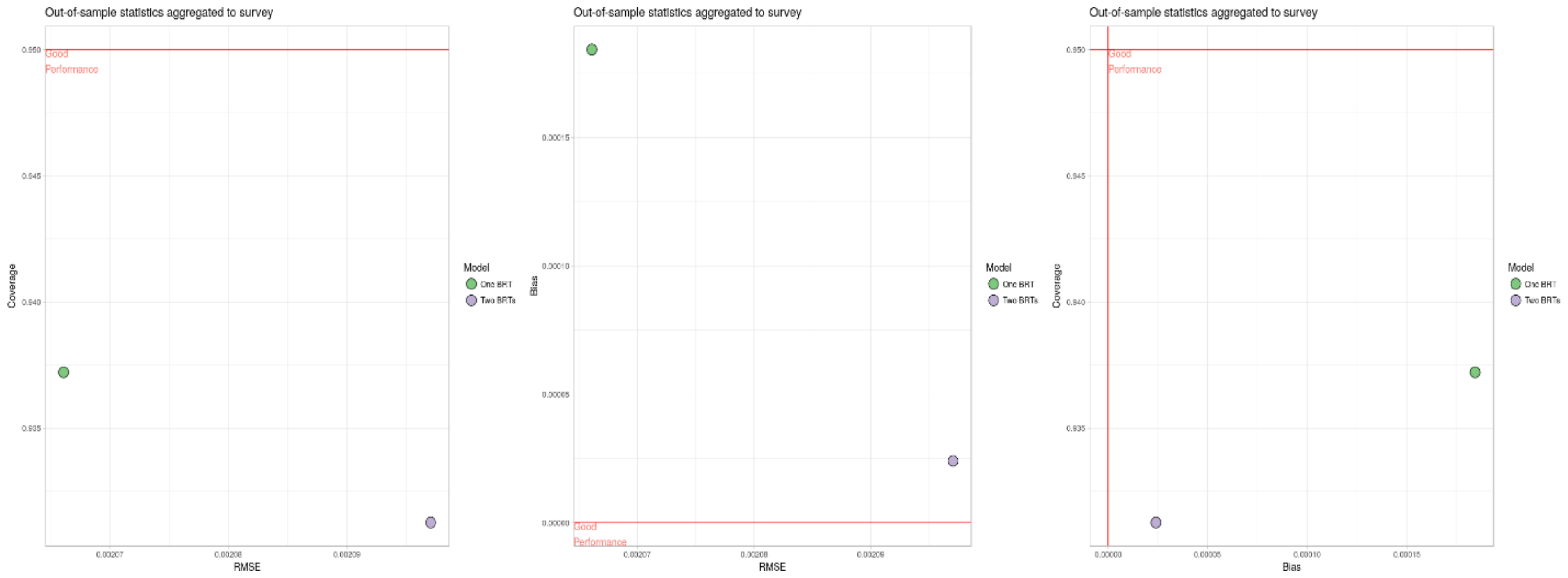

Supplementary Figure 18. Out of sample statistics of different model specifications

Coverage vs. RMSE, Bias vs. RMSE and Coverage vs. Bias are provided for five different model specifications with two different holdout schemes. Stacked + GP refers to the main approach used in this paper where a series of sub-models are ensembled using Gaussian Process Regression (GPR). GP refers to a GPR model with no covariates or sub-models. Covs refers to a standard linear regression using un-modelled covariates as inputs and with no Gaussian process. Raw + GP refers to a GPR model without the first stage stacking approach. Stacked covs refers to a basic linear regression as the ensembling mechanism rather than GPR. (**a**) Model performance when holdouts were created via a quad-tree algorithm and proportional to year. (**b**) Holdout scheme that involved randomly holding out entire surveys within a particular region.

a

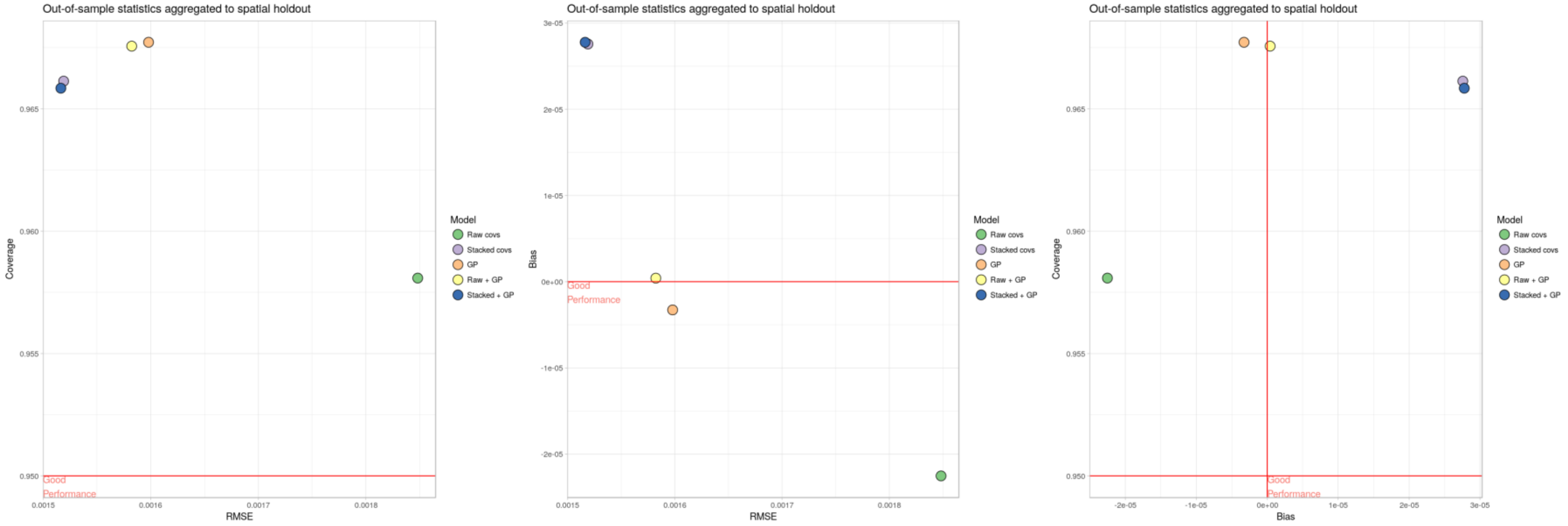

b

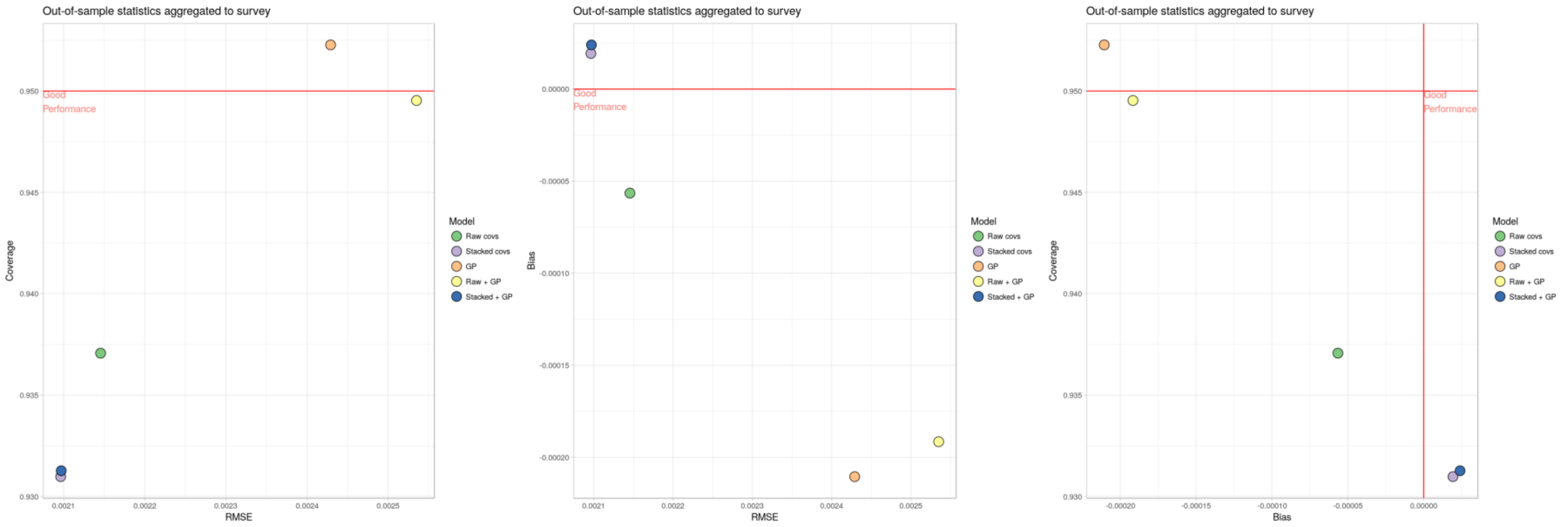

Out-of-sample statistics aggregated to survey
Coverage
RMSE
Good Performance
Model
Raw covs
Stacked covs
GP
Raw + GP
Stacked + GP
Out-of-sample statistics aggregated to survey
Bias
RMSE
Good Performance
Out-of-sample statistics aggregated to survey
Coverage
Bias
Good Performance

Supplementary Figure 19. Quad-tree folds for cross-validation

Visualisation of the spatial and temporal distribution of folds from the quadtree algorithm. Boxes indicate the spatial partitions generated by the quadtree algorithm, each of which contains data of a similar sample size. Time folds are 2000–2003, 2004–2007, 2008–2011, and 2012–2017, and are represented by box color.

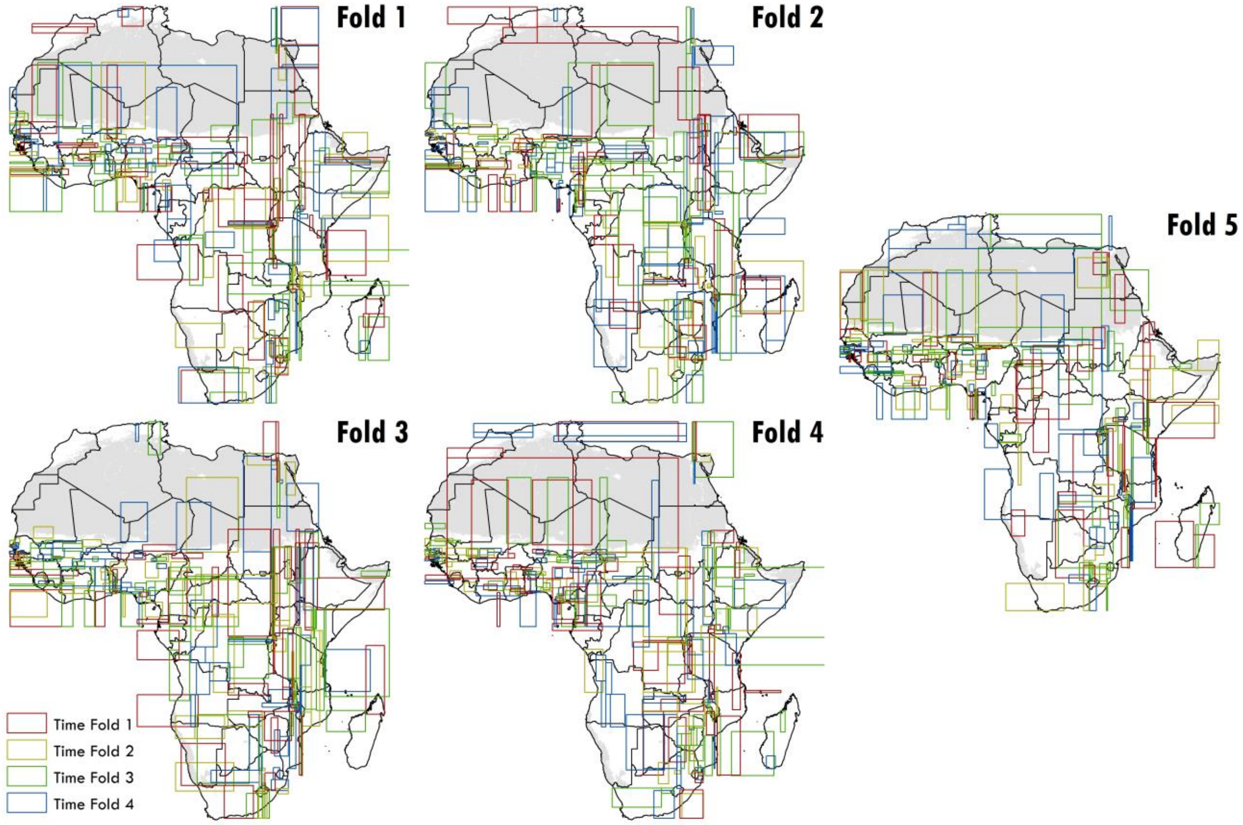

Supplementary Figure 20. Seasonal patterns in Lower Respiratory Infections

Fit of the seasonality sinusoidal regression for the North Africa and Middle East region (**a**) and fits for the regions within Sub-Saharan Africa (**b**).

a

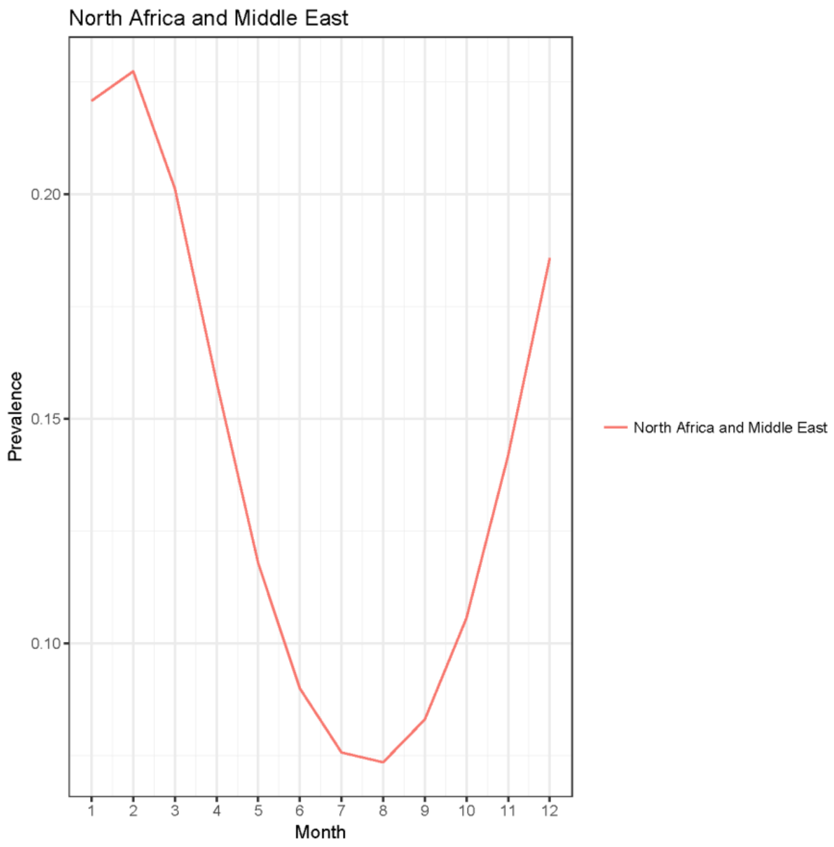

b

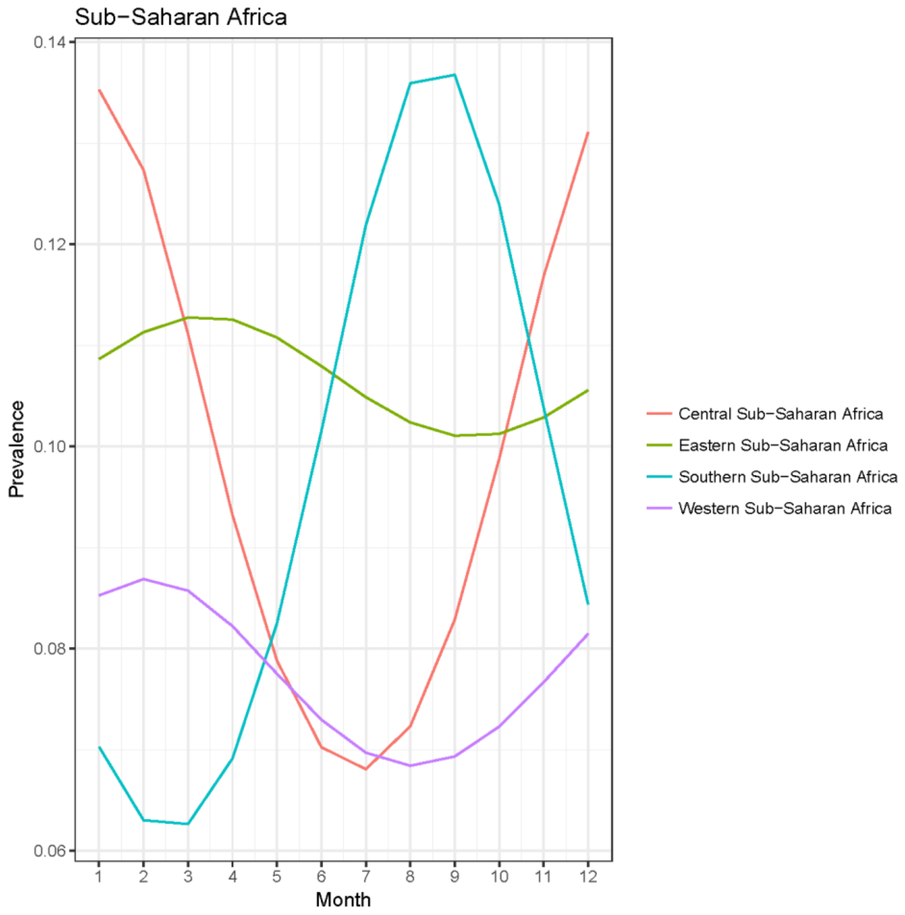

Sub−Saharan Africa
0.14
0.12
0.10
0.08
0.06
Prevalence
1
2
3
4
5
6
7
8
9
10
11
12
Month
Central Sub−Saharan Africa
Eastern Sub−Saharan Africa
Southern Sub−Saharan Africa
Western Sub−Saharan Africa

5.0 Supplementary Tables

Supplementary Table 1. Compliance for the Guidelines for Accurate and Transparent Health Estimates Reporting 43

Supplementary Table 2. Household surveys and censuses used in mapping 45

Supplementary Table 3. Covariates used in mapping 52

Supplementary Table 4. Spatial hyperparameter priors by region 55

Supplementary Table 5. Fitted parameters 56

Supplementary Table 6. Validation metrics for quad-tree spatial holdout scheme 57

Supplementary Table 7. Validation metrics for survey holdout scheme 58

Supplementary Table 8. Adjustment factors for standardising input survey data 59

Supplementary Table 9. INLA priors used in sensitivity analyses 60

Supplementary Table 10. Experiment 1 correlation coefficients, 2000-2017 61

Supplementary Table 11. Experiment 1 correlation coefficients by year, 2000-2017 62

Supplementary Table 12. Experiment 2 correlation coefficients, 2000-2017 63

Supplementary Table 13. Experiment 2 correlation coefficients by year, 2000-2017 64

Supplementary Table 1. Compliance for the Guidelines for Accurate and Transparent Health Estimates Reporting (GATHER)

Item #	Checklist item	Location reported
**Objectives and funding**		
**1**	Define the indicator(s), populations (including age, sex, and geographic entities), and time period(s) for which estimates were made.	Manuscript: Methods<br>Supplementary Information: Section 1.0, 2.0
**2**	List the funding sources for the work.	Manuscript: End notes
**Data Inputs**		
For all data inputs from multiple sources that are synthesised as part of the study:		
**3**	Describe how the data were identified and how the data were accessed.	Manuscript: Methods<br>Supplementary Information: Section 1.0, 2.0
**4**	Specify the inclusion and exclusion criteria. Identify all ad-hoc exclusions.	Manuscript: Methods<br>Supplementary Information: Section 1.0, 2.0
**5**	Provide information on all included data sources and their main characteristics. For each data source used, report reference information or contact name/institution, population represented, data collection method, year(s) of data collection, sex and age range, diagnostic criteria or measurement method, and sample size, as relevant.	Manuscript: Methods<br>Supplementary Information: Section 1.0, 2.0
**6**	Identify and describe any categories of input data that have potentially important biases (e.g., based on characteristics listed in item 5).	Supplementary Information: Section 2.0
For data inputs that contribute to the analysis but were not synthesised as part of the study:		
**7**	Describe and give sources for any other data inputs.	Manuscript: Methods<br>Supplementary Information: Section 2.0, 3.0
**8**	Provide all data inputs in a file format from which data can be efficiently extracted (e.g., a spreadsheet rather than a PDF), including all relevant meta-data listed in item 5. For any data inputs that cannot be shared because of ethical or legal reasons, such as third-party	Available at http://ghdx.healthdata.org/record/ihme-data/africa-under-5-lri-incidence-prevalence-

	ownership, provide a contact name or the name of the institution that retains the right to the data.	mortality-geospatial-estimates-2000-2017
**9**	Provide a conceptual overview of the data analysis method. A diagram may be helpful.	Supplementary Information: Supplementary Figure 1
**10**	Provide a detailed description of all steps of the analysis, including mathematical formulae. This description should cover, as relevant, data cleaning, data pre-processing, data adjustments and weighting of data sources, and mathematical or statistical model(s).	Manuscript: Methods<br>Supplementary Information: Sections 2.0
**11**	Describe how candidate models were evaluated and how the final model(s) were selected.	Manuscript: Methods<br>Supplementary Information: Sections 3.0, 4.0
**12**	Provide the results of an evaluation of model performance, if done, as well as the results of any relevant sensitivity analysis.	Manuscript: Methods<br>Supplementary Information: Sections 5.0
**13**	Describe methods for calculating uncertainty of the estimates. State which sources of uncertainty were, and were not, accounted for in the uncertainty analysis.	Manuscript: Methods<br>Supplementary Information: Sections 3.0
**14**	State how analytic or statistical source code used to generate estimates can be accessed.	https://github.com
**Results and Discussion**		
**15**	Provide published estimates in a file format from which data can be efficiently extracted.	Raster files for spatial data and CSVs of estimates are available at http://ghdx.healthdata.org/record/ihme-data/africa-under-5-lri-incidence-prevalence-mortality-geospatial-estimates-2000-2017
**16**	Report a quantitative measure of the uncertainty of the estimates (e.g., credible intervals).	Manuscript: Methods<br>Supplementary Information: Section 3.0
**17**	Interpret results in light of existing evidence. If updating a previous set of estimates, describe the reasons for changes in estimates.	Manuscript: Discussion, limitations, and future work
**18**	Discuss limitations of the estimates. Include a discussion of any modelling assumptions or data limitations that affect interpretation of the estimates.	Manuscript: Discussion, limitations, and future work

Supplementary Table 2. Household surveys and censuses used in mapping
Number identification (NID) can be used to locate a particular data source in the Global Health Data Exchange at http://ghdx.healthdata.org/record/ihme-data/africa-under-5-lri-incidence-prevalence-mortality-geospatial-estimates-2000-2017.

Country	Survey Start Year	Source	Number Identification (NID)	Children Sampled	Geo-positioned Clusters	Polygons
Algeria	2002	ARAB LEAGUE PAPFAM	627	3152	0	47
Algeria	2012	UNICEF MICS	210614	14701	0	7
Angola	2001	UNICEF MICS	687	5663	0	18
Angola	2015	DHS	218555	14313	625	0
Benin	2001	DHS	18950	5349	247	0
Benin	2006	DHS	18959	16075	0	12
Benin	2011	DHS	79839	13336	746	0
Benin	2014	UNICEF MICS	206075	12250	0	12
Botswana	2007	COUNTRY SPECIFIC	22125	2300	0	324
Burkina Faso	2003	DHS	19088	10575	397	0
Burkina Faso	2006	UNICEF MICS	1927	5217	195	0
Burkina Faso	2010	DHS	19133	14159	541	0
Burundi	2000	UNICEF MICS	1994	3325	0	17
Burundi	2005	UNICEF MICS	1981	6894	0	17
Burundi	2010	DHS	30431	7742	376	0
Burundi	2016	DHS	286766	13132	552	0
Cameroon	2000	UNICEF MICS	2053	3581	0	10
Cameroon	2004	DHS	19211	8085	461	0
Cameroon	2006	UNICEF MICS	2063	6362	0	191
Cameroon	2011	DHS	19274	11723	577	0
Cameroon	2014	UNICEF MICS	244455	7081	0	207
Central African Republic	2000	UNICEF MICS	2209	14271	0	17
Central African	2006	UNICEF MICS	2223	9585	0	16

Republic						
Central African Republic	2010	UNICEF MICS	82832	10474	0	17
Chad	2000	UNICEF MICS	2244	5384	0	15
Chad	2004	DHS	19315	5635	0	9
Chad	2010	UNICEF MICS	76701	17006	0	60
Chad	2014	DHS	157025	18623	624	0
Comoros	2000	UNICEF MICS	3114	4870	0	3
Comoros	2012	DHS	76850	2992	242	0
Congo	2005	DHS	19391	4835	0	12
Congo	2011	DHS	56151	9329	0	12
Côte d'Ivoire	2000	UNICEF MICS	26444	7943	0	11
Côte d'Ivoire	2006	UNICEF MICS	26433	8604	0	52
Côte d'Ivoire	2011	DHS	18533	7555	341	0
Democratic Republic of the Congo	2001	UNICEF MICS	3161	10254	0	11
Democratic Republic of the Congo	2007	DHS	19381	8779	293	0
Democratic Republic of the Congo	2010	UNICEF MICS	26998	10365	359	0
Democratic Republic of the Congo	2013	DHS	76878	17199	492	0
Djibouti	2002	ARAB LEAGUE PAPFAM	3392	1793	0	1
Djibouti	2006	UNICEF MICS	3404	2084	0	1
Djibouti	2006	UNICEF MICS	3404	2084	81	0
Egypt	2000	DHS	19511	11444	987	0
Egypt	2003	DHS	19529	5740	882	0
Egypt	2005	DHS	19521	13250	1288	0
Egypt	2008	DHS	26842	10677	1222	0
Egypt	2013	UNICEF MICS	159617	5090	0	6
Egypt	2014	DHS	154897	15744	1741	0

Equatorial Guinea	2000	UNICEF MICS	3655	2949	0	7
Eritrea	2002	DHS	19539	6366	0	6
Ethiopia	2000	DHS	19571	10031	533	0
Ethiopia	2005	DHS	19557	8871	528	0
Ethiopia	2008	COUNTRY SPECIFIC	26661	3846	0	11
Ethiopia	2010	DHS	21301	10434	571	0
Ethiopia	2016	DHS	218568	9618	622	0
Gabon	2000	DHS	19579	4405	0	40
Gabon	2012	DHS	76706	6019	330	0
Ghana	2003	DHS	19627	3824	408	0
Ghana	2006	UNICEF MICS	4694	3468	0	10
Ghana	2007	UNICEF MICS	160576	8457	0	4
Ghana	2008	DHS	21188	2942	401	0
Ghana	2010	UNICEF MICS	56241	250	51	0
Ghana	2011	UNICEF MICS	63993	6947	738	0
Ghana	2014	DHS	157027	5801	423	0
Guinea	2005	DHS	19683	6245	291	0
Guinea	2012	DHS	69761	7039	300	0
Guinea-Bissau	2000	UNICEF MICS	4808	5851	0	9
Guinea-Bissau	2006	UNICEF MICS	4818	5845	0	9
Guinea-Bissau	2014	UNICEF MICS	174049	7573	0	9
Kenya	2000	UNICEF MICS	7387	7235	814	0
Kenya	2003	DHS	20145	5925	399	0
Kenya	2007	UNICEF MICS	155335	955	78	0
Kenya	2008	UNICEF MICS	7401	14244	650	0
Kenya	2008	DHS	21365	6059	397	0
Kenya	2009	UNICEF MICS	56420	454	0	1
Kenya	2013	UNICEF MICS	203654	846	50	0
Kenya	2013	UNICEF MICS	203663	784	48	0
Kenya	2013	UNICEF MICS	203664	1052	56	0

Kenya	2014	DHS	157057	20851	1584	0
Lesotho	2000	UNICEF MICS	7721	1234	0	10
Lesotho	2004	DHS	20167	3486	379	0
Lesotho	2009	DHS	21382	3961	394	0
Lesotho	2014	DHS	157058	3138	397	0
Liberia	2006	DHS	20191	5704	291	0
Liberia	2013	DHS	77385	7606	322	0
Madagascar	2000	UNICEF MICS	27020	6562	0	6
Madagascar	2003	DHS	20223	5415	0	6
Madagascar	2008	DHS	21409	12228	585	0
Madagascar	2012	UNICEF MICS	125594	2997	127	0
Malawi	2000	DHS	20252	11926	559	0
Malawi	2004	DHS	20263	10904	520	0
Malawi	2006	UNICEF MICS	7919	22994	0	26
Malawi	2010	DHS	21393	19490	827	0
Malawi	2013	UNICEF MICS	161662	18981	0	31
Malawi	2015	DHS	218581	17280	850	0
Mali	2006	DHS	20274	14194	405	0
Mali	2009	UNICEF MICS	270627	23496	0	9
Mali	2012	DHS	77388	10326	413	0
Mali	2015	UNICEF MICS	248224	16202	0	8
Mauritania	2000	DHS	20322	4764	0	13
Mauritania	2007	UNICEF MICS	8115	8535	0	196
Mauritania	2011	UNICEF MICS	152783	9246	0	194
Mauritania	2015	UNICEF MICS	267343	10663	0	13
Morocco	2003	DHS	20361	6180	480	0
Mozambique	2003	DHS	20394	10326	0	11
Mozambique	2008	UNICEF MICS	27031	11452	0	11
Mozambique	2011	DHS	55975	11089	609	0
Namibia	2000	DHS	20417	3989	258	0
Namibia	2006	DHS	20428	5071	486	0
Namibia	2013	DHS	150382	5046	537	0
Niger	2000	UNICEF MICS	9439	5080	0	8

Niger	2006	DHS	20499	9193	0	8
Niger	2012	DHS	74393	12558	0	8
Nigeria	2003	DHS	20567	5989	359	0
Nigeria	2007	COUNTRY SPECIFIC	50426	1249	0	50
Nigeria	2007	UNICEF MICS	9516	16549	0	37
Nigeria	2008	DHS	21433	28647	886	0
Nigeria	2009	COUNTRY SPECIFIC	50441	925	0	48
Nigeria	2011	UNICEF MICS	76703	25192	0	37
Nigeria	2013	DHS	77390	31225	889	0
Nigeria	2016	UNICEF MICS	218613	28085	0	37
Rwanda	2000	UNICEF MICS	26930	3154	0	12
Rwanda	2000	DHS	20722	7922	0	12
Rwanda	2005	DHS	20740	8557	456	0
Rwanda	2007	DHS	21222	5428	246	0
Rwanda	2010	DHS	56040	9002	492	0
Rwanda	2014	DHS	157063	7856	492	0
São Tomé and Príncipe	2000	UNICEF MICS	27055	2208	0	4
São Tomé and Príncipe	2008	DHS	26866	1931	0	7
São Tomé and Príncipe	2014	UNICEF MICS	214640	2030	0	7
Senegal	2000	UNICEF MICS	27044	9064	0	10
Senegal	2005	DHS	26855	10624	366	0
Senegal	2010	DHS	56063	12103	385	0
Senegal	2012	DHS	111432	6862	200	0
Senegal	2014	DHS	191270	6842	0	14
Senegal	2015	DHS	218592	6935	214	0
Senegal	2016	DHS	286772	6725	214	0
Sierra Leone	2000	UNICEF MICS	11639	2704	0	4
Sierra Leone	2005	UNICEF MICS	11649	5246	0	14
Sierra Leone	2008	DHS	21258	5590	349	0
Sierra Leone	2010	UNICEF MICS	76700	8598	0	14
Sierra Leone	2013	DHS	131467	11938	435	0

Somalia	2006	UNICEF MICS	11774	6305	0	18
Somalia	2011	UNICEF MICS	91507	4408	258	0
Somalia	2011	UNICEF MICS	91508	4714	276	0
South Africa	2002	COUNTRY SPECIFIC	115481	9576	0	9
South Africa	2004	COUNTRY SPECIFIC	11788	8631	0	9
South Africa	2005	COUNTRY SPECIFIC	11789	10233	0	9
South Africa	2006	COUNTRY SPECIFIC	115486	9876	0	9
South Africa	2007	COUNTRY SPECIFIC	11790	10834	0	9
South Africa	2008	COUNTRY SPECIFIC	115488	9552	0	9
South Africa	2009	COUNTRY SPECIFIC	115489	9961	0	9
South Africa	2010	COUNTRY SPECIFIC	115490	10266	0	9
South Africa	2011	COUNTRY SPECIFIC	115491	9589	0	9
South Africa	2012	COUNTRY SPECIFIC	135534	9509	0	9
South Africa	2013	COUNTRY SPECIFIC	162652	9799	0	9
South Sudan	2000	UNICEF MICS	12232	1399	0	46
South Sudan	2010	UNICEF MICS	32189	8338	0	10
Sudan	2000	UNICEF MICS	12243	23295	0	16
Sudan	2010	UNICEF MICS	153643	13282	0	15
Sudan	2014	UNICEF MICS	200617	14081	0	18
Swaziland	2000	UNICEF MICS	12320	3509	0	4
Swaziland	2006	DHS	20829	2761	270	0
Swaziland	2010	UNICEF MICS	30325	2647	0	4
Swaziland	2014	UNICEF MICS	200707	2693	0	4
Tanzania	2004	DHS	20875	8564	0	26
Tanzania	2009	DHS	21331	7750	458	0
Tanzania	2015	DHS	218593	10226	608	0
The Gambia	2000	UNICEF MICS	3922	3632	0	8
The Gambia	2005	UNICEF MICS	3935	6543	0	37
The Gambia	2013	DHS	77384	8088	0	37
Togo	2000	UNICEF MICS	12886	3129	0	6
Togo	2006	UNICEF MICS	12896	4074	0	6
Togo	2010	UNICEF MICS	40021	4746	0	6
Togo	2013	DHS	77515	6979	330	0

Tunisia	2001	ARAB LEAGUE PAPFAM	12978	1820	0	24
Tunisia	2011	UNICEF MICS	76709	2899	0	9
Uganda	2000	DHS	20993	6309	270	0
Uganda	2006	DHS	21014	7657	336	0
Uganda	2011	DHS	56021	7795	400	0
Zambia	2001	DHS	21102	6877	0	72
Zambia	2007	DHS	21117	6401	319	0
Zambia	2008	COUNTRY SPECIFIC	26702	3711	0	9
Zambia	2013	DHS	77516	13412	719	0
Zimbabwe	2005	DHS	21163	5204	396	0
Zimbabwe	2009	UNICEF MICS	35493	7242	0	10
Zimbabwe	2010	DHS	55992	5372	393	0
Zimbabwe	2014	UNICEF MICS	152720	9884	0	10
Zimbabwe	2015	DHS	157066	6128	399	0

Supplementary Table 3. Covariates used in mapping

A variety of socioeconomic and environmental variables were used to predict LRI prevalence. Where available, the finest spatio-temporal resolution of gridded data sets was used.

Covariate	Temporal Resolution	Source	Reference
Average daily mean temperature	Annual	CRUTS	Harris, I., Jones, P. d., Osborn, T. j. & Lister, D. h. Updated high-resolution grids of monthly climatic observations – the CRU TS3.10 dataset. Int. J. Climatol. 34, 623–642 (2014).<br><br>University of East Anglia. Climatic Research Unit TS v. 3.24 dataset. Available at: https://crudata.uea.ac.uk/cru/data/hrg/cru_ts_3.24.01/. (Accessed: 24th July 2017).
Elevation	Static	NOAA	Hastings, David A., and Paula K. Dunbar. Global Land One-kilometer Base Elevation (GLOBE) Digital Elevation Model, Documentation, Volume 1.0. Key to Geophysical Records Documentation (KGRD) 34. National Oceanic and Atmospheric Administration, National Geophysical Data Center, 325 Broadway, Boulder, Colorado 80303, U.S.A (1999).<br><br>GLOBE Task Team and others (Hastings, David A., Paula K. Dunbar, Gerald M. Elphingstone, Mark Bootz, Hiroshi Murakami, Hiroshi Maruyama, Hiroshi Masaharu, Peter Holland, John Payne, Nevin A. Bryant, Thomas L. Logan, J.-P. Muller, Gunter Schreier, and John S. MacDonald), eds., 1999. The Global Land One-kilometer Base Elevation (GLOBE) Digital Elevation Model, Version 1.0. National Oceanic and Atmospheric Administration, National Geophysical Data Center, 325 Broadway, Boulder, Colorado 80303, U.S.A. Available at: https://www.ngdc.noaa.gov/mgg/topo/globe.html. (Accessed: 16th February

			2017)
Enhanced Vegetation Index (EVI)	Annual	MODIS	Huete, A., Justice, C. & van Leeuwen, W. MODIS vegetation index (MOD 13) algorithm theoretical basis document. (1999).<br><br>USGS & NASA. Vegetation indices 16-Day L3 global 500m MOD13A1 dataset. Available at: https://lpdaac.usgs.gov/dataset_discovery/modis/modis_products_table/mod13a1. (Accessed: 25th July 2017)<br><br>Weiss, D. J. et al. An effective approach for gap-filling continental scale remotely sensed time-series. Isprs J. Photogramm. Remote Sens. 98, 106–118 (2014).
Maternal education	Annual	Internally modelled	Graetz N, Friedman J, Osgood-Zimmerman A, Burstein R, Biehl MH, Shields C, Mosser JF, Casey DC, Deshpande A, Earl L, Reiner RC, Ray SE, Fullman N, Levine AJ, Stubbs RW, Mayala BK, Longbottom J, Browne AJ, Bhatt S, Weiss DJ, Gething PW, Mokdad AH, Lim SS, Murray CJLM, Gakidou E, Hay SI. Mapping local variation in educational attainment across Africa. Nature. 28 Feb 2018. doi:10.1038/nature25761
Night time lights	Annual	NOAA DMSP	Savory et al. Intercalibration and Gaussian Process Modeling of Nighttime Lights Imagery for Measuring Urbanisation Trends in Africa 2000– 2013. Remote Sens. 9, (2017).
Outdoor air pollution (PM2.5 concentration)	Annual	Global Burden of Disease study	Global Burden of Disease Collaborative Network. Global Burden of Disease Study 2016 (GBD 2016) Covariates 1980-2016. Seattle, United States: Institute for Health Metrics and Evaluation (IHME), 2017.<br><br>Institute for Health Metrics and Evaluation. Global Health Data Exchange. Available at: http://internal-ghdx.healthdata.org/gbd-2016/data-input-sources.
Prevalence of Malaria in	Annual	Malaria Atlas Project	Bhatt, S., Weiss, D. J. et al. The effect of malaria control on Plasmodium falciparum in Africa between 2000 and 2015. Nature 526, pages 207–211 (08

Africa			October 2015) doi:10.1038/nature15535
Sanitation	Annual	Global Burden of Disease study	Global Burden of Disease Collaborative Network. Global Burden of Disease Study 2016 (GBD 2016) Covariates 1980-2016. Seattle, United States: Institute for Health Metrics and Evaluation (IHME), 2017.<br><br>Institute for Health Metrics and Evaluation. Global Health Data Exchange. Available at: http://internal-ghdx.healthdata.org/gbd-2016/data-input-sources.
Stunting		Internally modelled	Osgood-Zimmerman A, Millear AI, Stubbs RW, Shields C, Pickering BV, Earl L, Graetz N, Kinyoki DK, Ray SE, Bhatt S, Browne AJ, Burstein R, Cameron E, Casey DC, Deshpande A, Fullman N, Gething PW, Gibson HS, Henry NJ, Herrero M, Krause LK, Letourneau ID, Levine AJ, Liu PY, Longbottom J, Mayala BK, Mosser JF, Noor AM, Pigott DM, Piwoz EG, Rao P, Rawat R, Reiner RC, Smith DL, Weiss DJ, Wiens KE, Mokdad AH, Lim SS, Murray CJL, Kassebaum NJ, Hay SI. Mapping child growth failure in Africa between 2000 and 2015. Nature. 28 Feb 2018. doi:10.1038/nature25760
Travel time to nearest settlement	Static	MODIS	Weiss, D. J. et al. A global map of travel time to cities to assess inequalities in accessibility in 2015. doi:10.1038/nature25181 (2018).
Vaccine coverage - PCV3	Annual	Internally modelled	Lozano, R., et al. "Measuring progress from 1990 to 2017 and projecting attainment to 2030 of the health-related Sustainable Development Goals for 195 countries and territories: a systematic analysis for the Global Burden of Disease Study 2017." The Lancet 392.10159 (2018): 2091-2138.
Vaccine coverage - HIB	Annual	Internally modelled	Lozano, R., et al. "Measuring progress from 1990 to 2017 and projecting attainment to 2030 of the health-related Sustainable Development Goals for 195 countries and territories: a systematic analysis for the Global Burden of Disease Study 2017." The Lancet 392.10159 (2018): 2091-2138.

Supplementary Table 4. Spatial hyperparameter priors by region

Region	μ_{θ_1}	$\sigma^2_{\theta_1}$	μ_2	$\sigma^2_{\theta_2}$
**Central sub-Saharan Africa**	-0.23082	10	-1.03469	10
**Eastern sub-Saharan Africa**	0.104454	10	-1.36997	10
**Northern Africa**	0.22028	10	-1.48579	10
**Southern sub-Saharan Africa**	-0.17385	10	-1.09166	10
**Western sub-Saharan Africa**	0.181774	10	-1.44729	10

Supplementary Table 5. Fitted parameters

Posterior lower, median, and upper quantiles (0.025%, 0.50%, 0.975%) for the main parameters by region. The first four rows provide information on the fixed effects: the intercept (int) and the covariates (gam, gbm, and enet) corresponding to the predicted ensemble rasters. Fitted values for the spatio-temporal field hyperparameters and the precision parameters (inverse variance) for random effects are shown in the bottom four rows.

	Central sub-Saharan Africa quantiles			Eastern sub-Saharan Africa quantiles			Northern Africa quantiles			Southern sub-Saharan Africa quantiles			Western sub-Saharan Africa quantiles		
	**0.025**	**0.500**	**0.975**	**0.025**	**0.500**	**0.975**	**0.025**	**0.500**	**0.975**	**0.025**	**0.500**	**0.975**	**0.025**	**0.500**	**0.975**
**int**	-0.227	-0.101	0.022	-0.202	-0.113	-0.026	-0.241	-0.125	-0.011	-0.428	-0.252	-0.080	-0.220	-0.097	0.026
**gam**	-0.517	0.011	0.540	-0.394	-0.099	0.199	-0.325	0.033	0.390	-0.427	0.435	1.312	-0.276	0.021	0.323
**gbm1**	-0.756	-0.257	0.241	-0.660	-0.389	-0.120	-0.574	-0.300	-0.027	-1.875	-0.976	-0.089	-0.715	-0.422	-0.130
**gbm2**	0.791	1.144	1.493	1.190	1.411	1.629	0.942	1.223	1.501	1.028	1.469	1.900	1.018	1.242	1.463
**enet**	0.026	0.101	0.180	0.040	0.077	0.115	0.001	0.045	0.092	-0.007	0.073	0.156	0.114	0.158	0.203
**Nominal Range**	0.551	2.957	7.029	1.562	3.576	6.997	1.324	3.181	6.347	0.163	2.507	6.740	1.649	3.538	6.576
**Nominal Variance**	-0.761	1.919	6.860	-2.896	0.718	5.643	-2.526	0.322	4.542	-1.917	1.422	6.033	-3.394	-2.002	-0.775
**Ar1 ϱ**	-0.988	-0.035	0.986	-1.000	-0.434	0.982	-0.929	0.074	0.982	-0.991	-0.290	0.930	-0.885	0.005	0.916
**Precision for Country random effect**	12.285	17.245	24.340	22.733	29.718	38.333	13.109	18.058	25.679	10.438	15.212	21.751	20.073	26.415	34.709

Supplementary Table 6. Validation metrics for quad-tree spatial holdout scheme

Statistics calculated by quad-tree leaf (n = 447) and averaged by period. Pearson correlations are shown.

Sample	Spatial Holdout	Period	Mean Err.	RMSE	Corr.	95% Cov.
In Sample	Quad-tree	2000–2003	0.00003	0.00107	0.9	97.63
In Sample	Quad-tree	2004–2007	-0.00028	0.00099	0.91	98.94
In Sample	Quad-tree	2008–2011	-0.00014	0.00081	0.92	98.61
In Sample	Quad-tree	2012–2017	-0.00009	0.00083	0.94	98.62
In Sample	Quad-tree	2000–2017	-0.00012	0.00091	0.93	98.43
Out of Sample	Quad-tree	2000–2003	0.00049	0.00187	0.66	94.65
Out of Sample	Quad-tree	2004–2007	-0.00016	0.00157	0.74	96.7
Out of Sample	Quad-tree	2008–2011	-0.00013	0.00124	0.78	97.3
Out of Sample	Quad-tree	2012–2017	-0.00002	0.00142	0.79	97.18
Out of Sample	Quad-tree	2000–2017	0.00003	0.00152	0.77	96.44

Supplementary Table 7. Validation metrics for survey holdout scheme

Statistics calculated by survey (n = 191) and averaged by period. Pearson correlations are shown.

Sample	Spatial Holdout	Period	Mean Err.	RMSE	Corr.	95% Cov.
In Sample	Survey	2000–2003	0.00014	0.001	0.9	97.6
In Sample	Survey	2004–2007	-0.00032	0.00072	0.95	98.8
In Sample	Survey	2008–2011	-0.00017	0.00045	0.97	98.8
In Sample	Survey	2012–2017	-0.00012	0.00063	0.96	98.6
In Sample	Survey	2000–2017	-0.00012	0.00071	0.95	98.48
Out of Sample	Survey	2000–2003	0.00061	0.00258	0.18	89.61
Out of Sample	Survey	2004–2007	-0.00025	0.00163	0.7	94.72
Out of Sample	Survey	2008–2011	-0.00026	0.00165	0.48	95.08
Out of Sample	Survey	2012–2017	0.00006	0.00237	0.08	92.78
Out of Sample	Survey	2000–2017	0.00002	0.0021	0.45	93.2

Supplementary Table 8. Adjustment factors for standardising input survey data

Definition	Adjustment for missing fever[20]	Adjustment for missing chest symptoms	Adjustment for self-reported data	Overall adjustment
Fever with cough and difficulty breathing & symptoms in chest	None	None	0.25 (0.23–0.26)	0.25 [21]
Fever with cough and difficulty breathing	None	0.58 (0.55–0.63)	0.25 (0.23–0.26)	0.145 [20]
Cough and difficulty breathing & symptoms in chest	0.8	None	0.25 (0.23–0.26)	0.2
Cough and difficulty breathing	0.58	0.58 (0.55–0.63)	0.25 (0.23–0.26)	0.084

[14] Rambaud-Althaus et al. (2015) found that fever was present in 94% of pneumonia episodes, and that accuracy of difficulty breathing was 12%

[15] Hazir et al. (2013) found that the accuracy of DHS suspected pneumonia + fever was between 5.1 and 33.9% for clinician-diagnosed pneumonia in Bangladesh and Pakistan and the specificity was 63.8–85.7%

Supplementary Table 9. INLA priors used in sensitivity analyses

Parameter	Base specification	Experiment 1	Experiment 2
nugget_prior	$loggamma(\alpha = 2, \gamma = 1)$	$loggamma(\alpha = 4, \gamma = 1)$	$loggamma(\alpha = 1, \gamma = 1)$
country_re_prior	$loggamma(\alpha = 2, \gamma = 1)$	$loggamma(\alpha = 4, \gamma = 1)$	$loggamma(\alpha = 1, \gamma = 1)$
temporal_model_theta_prior	$loggamma(\alpha = 1, \gamma = 5 \times 10^{-5})$	$loggamma(\alpha = 2, \gamma = 5 \times 10^{-5})$	$loggamma(\alpha = 0.5, \gamma = 5 \times 10^{-5})$
rho_prior	$N(\mu = 0, \sigma^2 = \frac{1}{2.58^2})$	$N(\mu = 0, \sigma^2 = \frac{2}{2.58^2})$	$N(\mu = 0, \sigma^2 = \frac{0.5}{2.58^2})$

Supplementary Table 10. Experiment 1 correlation coefficients, 2000–2017

Experiment 1 vs. base specification: Pearson correlation coefficients for relationship between first-level administrative subdivision prevalence means, 2000–2017.

**Region**	**Correlation coefficient**
Central Sub-Saharan Africa (n = 88 units)	0.9988
Eastern Sub-Saharan Africa (n = 265 units)	0.9992
Northern Africa (n = 154 units)	0.9943
Southern Sub-Saharan Africa (n = 62 units)	0.9966
Western Sub-Saharan Africa (n = 212 units)	0.9977

Supplementary Table 11. Experiment 1 correlation coefficients by year, 2000–2017

Experiment 1 vs. base specification: Pearson correlation coefficients for relationship between first-level administrative subdivision prevalence means by year.

Region	Year	Correlation coefficient
Central Sub-Saharan Africa (n = 88 units)	2000	0.9984
	2005	0.9984
	2010	0.9981
	2015	0.9975
Eastern Sub-Saharan Africa (n = 265 units)	2000	0.9987
	2005	0.9993
	2010	0.9987
	2015	0.9985
Northern Africa (n = 154 units)	2000	0.9846
	2005	0.9760
	2010	0.9918
	2015	0.9950
Southern Sub-Saharan Africa (n = 62 units)	2000	0.9992
	2005	0.9975
	2010	0.9980
	2015	0.9950
Western Sub-Saharan Africa (n = 212 units)	2000	0.9962
	2005	0.9957
	2010	0.9971
	2015	0.9992

Supplementary Table 12. Experiment 2 correlation coefficients, 2000–2017

Experiment 2 vs. base specification: Pearson correlation coefficients for relationship between first-level administrative subdivision prevalence means, 2000–2017.

Region	Correlation coefficient
Central Sub-Saharan Africa (n = 88 units)	0.9976
Eastern Sub-Saharan Africa (n = 265 units)	0.9994
Northern Africa (n = 154 units)	0.9853
Southern Sub-Saharan Africa (n = 62 units)	0.9974
Western Sub-Saharan Africa (n = 212 units)	0.9976

Supplementary Table 13. Experiment 2 correlation coefficients by year, 2000–2017

Experiment 2 vs. base specification: Pearson correlation coefficients for relationship between admin 1 prevalence means by year.

Region	Year	Correlation coefficient
Central Sub-Saharan Africa (n = 88 units)	2000	0.9976
	2005	0.9957
	2010	0.9984
	2015	0.9889
Eastern Sub-Saharan Africa (n = 265 units)	2000	0.9990
	2005	0.9994
	2010	0.9993
	2015	0.9991
Northern Africa (n = 154 units)	2000	0.9692
	2005	0.9393
	2010	0.9050
	2015	0.9881
Southern Sub-Saharan Africa (n = 62 units)	2000	0.9992
	2005	0.9972
	2010	0.9979
	2015	0.9934
Western Sub-Saharan Africa (n = 212 units)	2000	0.9956
	2005	0.9957
	2010	0.9963
	2015	0.9993

6.0 Supplementary Information References

1. GBD 2016 Lower Respiratory Infections Collaborators. Estimates of the global, regional, and national morbidity, mortality, and aetiologies of lower respiratory infections in 195 countries, 1990-2016: a systematic analysis for the Global Burden of Disease Study 2016. Lancet Infect Dis 2018;18(11):1191–210.

2. Flaxman AD, Voz T, Murray CJLM. An integrative metaregression framework for descriptive epidemiology. University of Washington Press; 2015.

3. Kish L. Survey sampling. New York, London: John Wiley & Sons, Inc.; 1965.

4. Worldpop: get data [Internet]. [cited 2018 Jun 13];Available from: http://www.worldpop.org.uk/data/get_data/

5. Bhatt S, Cameron E, Flaxman SR, Weiss DJ, Smith DL, Gething PW. Improved prediction accuracy for disease risk mapping using Gaussian process stacked generalization. J R Soc Interface 2017;14(134):20170520.

6. Stein ML. Interpolation of Spatial Data: Some Theory for Kriging [Internet]. Springer New York; 1999 [cited 2017 Oct 17]. Available from: //www.springer.com/us/book/9780387986296

7. Gelfand AE, Diggle P, Guttorp P, Fuentes M, editors. Handbook of Spatial Statistics. 1 edition. Boca Raton: CRC Press; 2010.

8. Rue H, Martino S, Chopin N. Approximate Bayesian inference for latent Gaussian models by using integrated nested Laplace approximations. J R Stat Soc Ser B Stat Methodol 2009;71(2):319–92.

9. Martins TG, Simpson D, Lindgren F, Rue H. Bayesian computing with INLA: New features. Comput Stat Data Anal 2013;67:68–83.

10. Lidngren F, Rue H, Lindström J. An explicit link between Gaussian fields and Gaussian Markov random fields: the stochastic partial differential equation approach. J R Stat Soc Ser B Stat Methodol 2011;73(4):423–98.

11. GBD 2017 Causes of Death Collaborators. Global, regional, and national age-sex-specific mortality for 282 causes of death in 195 countries and territories, 1980-2017: a systematic analysis for the Global Burden of Disease Study 2017. Lancet Lond Engl 2018;392(10159):1736–88.

12. Feikin DR, Scott JAG, Gessner BD. Use of vaccines as probes to define disease burden. Lancet Lond Engl 2014;383(9930):1762–70.

13. O'Brien KL, Wolfson LJ, Watt JP, et al. Burden of disease caused by Streptococcus pneumoniae in children younger than 5 years: global estimates. Lancet Lond Engl 2009;374(9693):893–902.

14. Johnson HL, Deloria-Knoll M, Levine OS, et al. Systematic evaluation of serotypes causing invasive pneumococcal disease among children under five: the pneumococcal global serotype project. PLoS Med 2010;7(10).

15. Swingler G, Fransman D, Hussey G. Conjugate vaccines for preventing Haemophilus influenzae type B infections. Cochrane Database Syst Rev 2007;(2):CD001729.

16. Lucero MG, Dulalia VE, Nillos LT, et al. Pneumococcal conjugate vaccines for preventing vaccine-type invasive pneumococcal disease and X-ray defined pneumonia in children less than two years of age. Cochrane Database Syst Rev 2009;(4):CD004977.

17. World Health Organization, UNICEF. Ending preventable child deaths from pneumonia and diarrhoea by 2025: The integrated Global Action Plan for Pneumonia and Diarrhoea (GAPPD). 2013.

18. Ord JK, Getis A. Local Spatial Autocorrelation Statistics: Distributional Issues and an Application. Geogr Anal 1995;27(4):286–306.

19. GBD 2017 Disease and Injury Incidence and Prevalence Collaborators. Global, regional, and national incidence, prevalence, and years lived with disability for 354 diseases and injuries for 195 countries and territories, 1990-2017: a systematic analysis for the Global Burden of Disease Study 2017. Lancet 2018;392(10159):1789–858.

20. Rambaud-Althaus C, Althaus F, Genton B, D'Acremont V. Clinical features for diagnosis of pneumonia in children younger than 5 years: a systematic review and meta-analysis. Lancet Infect Dis 2015;15(4):439–50.

21. Hazir T, Begum K, el Arifeen S, et al. Measuring Coverage in MNCH: A Prospective Validation Study in Pakistan and Bangladesh on Measuring Correct Treatment of Childhood Pneumonia. PLoS Med [Internet] 2013 [cited 2018 Jun 13];10(5). Available from: https://www.ncbi.nlm.nih.gov/pmc/articles/PMC3646205/
